# Tumor microenvironment penetrating chitosan nanoparticles for elimination of cancer relapse and minimal residual disease

**DOI:** 10.3389/fonc.2022.1054029

**Published:** 2022-11-30

**Authors:** Hossein Mahmudi, Mohammad Amin Adili-Aghdam, Mohammad Shahpouri, Mehdi Jaymand, Zohreh Amoozgar, Rana Jahanban-Esfahlan

**Affiliations:** ^1^ Department of Medical Biotechnology, Faculty of Advanced Medical Sciences, Tabriz University of Medical Sciences, Tabriz, Iran; ^2^ Student Research Committee, Tabriz University of Medical Sciences, Tabriz, Iran; ^3^ Research Center for Pharmaceutical Nanotechnology, Biomedicine Institute, Tabriz University of Medical Sciences, Tabriz, Iran; ^4^ Nano Drug Delivery Research Center, Health Technology Institute, Kermanshah University of Medical Sciences, Kermanshah, Iran; ^5^ Edwin L. Steele Laboratories, Department of Radiation Oncology, Massachusetts General Hospital and Harvard Medical School, Boston, MA, United States; ^6^ Stem Cell Research Center, Tabriz University of Medical Sciences, Tabriz, Iran

**Keywords:** chitosan, tumor heterogeneity, tumor microenvironment, recurrent cancer, drug delivery, minimal residual disease

## Abstract

Chitosan and its derivatives are among biomaterials with numerous medical applications, especially in cancer. Chitosan is amenable to forming innumerable shapes such as micelles, niosomes, hydrogels, nanoparticles, and scaffolds, among others. Chitosan derivatives can also bring unprecedented potential to cross numerous biological barriers. Combined with other biomaterials, hybrid and multitasking chitosan-based systems can be realized for many applications. These include controlled drug release, targeted drug delivery, post-surgery implants (immunovaccines), theranostics, biosensing of tumor-derived circulating materials, multimodal systems, and combination therapy platforms with the potential to eliminate bulk tumors as well as lingering tumor cells to treat minimal residual disease (MRD) and recurrent cancer. We first introduce different formats, derivatives, and properties of chitosan. Next, given the barriers to therapeutic efficacy in solid tumors, we review advanced formulations of chitosan modules as efficient drug delivery systems to overcome tumor heterogeneity, multi-drug resistance, MRD, and metastasis. Finally, we discuss chitosan NPs for clinical translation and treatment of recurrent cancer and their future perspective.

## Graphical Abstract

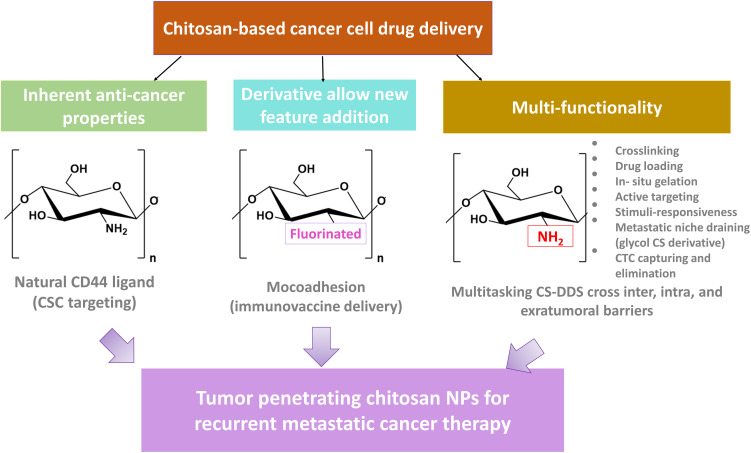

## 1 Introduction

Despite all advancements in drug delivery strategies developed for targeting solid tumors, efficacy is still suboptimal due to numerous biological barriers, including endothelial, cell membrane, organelle membrane, and the nanoparticle’s ability for on-target/off-target recognition, penetration, localization, concentration, and homogenous distribution. Most of these bottlenecks for successful NP delivery originate from the nature of the solid tumor microenvironment (TME), including the ability of immune editing, hypoxia, cancer stem cells (CSCs), extracellular matrix (ECM) components, and heterogeneity, among others (1, 2). TME leverages inherent resistance mechanisms such as hypoxia and pH gradients and acquired resistance mechanisms such as clonal evolution and heterogeneity after surgery or chemotherapy. These mechanisms promote a rare population of resistant tumor cells with properties of cancer stem-like cells (CSLS), which survive therapy and promote tumorigenesis, relapse, and metastasis (3, 4).

We often use natural products to develop drugs, scaffolds, and nanodrug delivery systems (NDDS) for biomedical applications and human clinical trials. Chitosan (CS) is the second most abundant polysaccharide after cellulose. CS is a biocompatible and biodegradable long copolymer of glucosamine and *N*-acetyl glucosamine as it breaks down into harmless and readily absorbed amino sugars (5). Alkaline deacetylation of chitin polysaccharides—ranging from 70% to 85%—produces CS with an average molecular weight of 3.8 to 500 kDa, and can be digested by lysozyme. CS is the only polysaccharide with a positive charge due to amine groups (5).

CS is widely studied for NDDS because of its biocompatibility, low cost, nontoxicity, low immunogenicity, and biodegradability. An added advantage of CS is functionalizability with multiple moieties and other polymers. Therefore, we can build multitasking platforms, including hybrid, theranostics, and multimodal systems for efficient cancer treatment (6, 7). Also, CS’s poor solubility in aqueous solutions above pH 6 promoted the development of various CS derivatives with improved solubility over a broad pH range. As each derivative is functionalized with a specific moiety such as carboxyl, fluorinated, quaternized, glycosylated, methyl, and others, this modification has far broadened the many applications. The broad application has made a CS the biomaterial of choice for numerous purposes, especially for efficient drug delivery by crossing a variety of biological barriers and on-demand drug release to overcome the heterogeneous nature of solid tumors, with the capability to avoid cancer relapse by targeting residual tumor cells lingering after therapy/surgery (8).

In this review, we aim to highlight the potential of CS and its derivatives as a promising biomaterial to meet numerous biomedical demands, with a particular emphasis on its capability to build advanced enabling NDDS platforms to cross multiple biological barriers, including blood, membrane, and TME-related obstacles and for the treatment of minimal residual disease (MRD) and recurrent cancer. To this end, we first looked into chitosan’s inherent properties and its different derivatives. Then, we discussed other formats and implications of CS-derived NDDS for targeting and eliminating recurrence. Finally, we included an overview of the clinical translation of CS NPs for the treatment of recurrent cancer.

## 2 Chitosan: Inherent properties

Chitosan is a linear polysaccharide derived by the deacetylation of the *N*-acetyl glucosamine units of chitin, a natural polymer from crustacean shells, through hydrolysis at high temperatures under alkaline conditions. Chitosan is abundant in nature and possesses inherent properties that render it appealing to use as a drug delivery system. We will highlight the critical features of chitosan in the following section.

### 2.1 Antibacterial

Chitosan-based biomaterials possess superior antimicrobial and antifungal activity against clinically important antibiotic-resistant pathogens, such as *E. coli* and MERS. As a cationic agent, chitosan binds to negatively charged bacteria membrane and increases membrane permeability, resulting in leakage of intracellular components and, finally, cell death (9, 10). Notably, unlike tested antibiotics used in human and veterinary medicine, there was no observation for developing resistant mutants in serial passage assays over a long period of more than 15 days (11).

### 2.2 Biocompatibility, biodegradability, and pH responsiveness

pH changes can degrade chitosan NPs. Meanwhile, functionalization can increase CS stability. In this line, the pH responsiveness of CS allows for the formulation of biodegradable yet controlled and sustained release of cargoes. Thus, biodegradable static forms are synthesized through physical ionic gelation of CS using a polyanionic agent such as TPP (12). Meanwhile, a dynamic or non-biodegradable form of CS generated through chemical cross-linking allows for repeated and regenerative potential with controlled swelling and shrinkage performance of hydrogel in response to biochemical changes in their environment. The latter feature can be highly appreciated for tissue regeneration and treatment of chronic disease and can be formulated for systemic or local (drug depot) purposes (9).

### 2.3 Drug reservoir and controlled release/local treatment

Chitosan can be used as an efficient drug reservoir for the codelivery of various hydrophilic and hydrophobic cargoes. Complexation with nucleic acids such as miRNA, siRNA, plasmid DNA, and CpG ODN can be achieved by simple electrostatic adsorption (10). CS DDSs can accommodate different types of cargoes loaded, including natural, synthetic, and semi-synthetic vesicular particles such as niosomes, liposomes, and different cell types (CAR T cells, stem cells, dendrimers, gold NPs, photosensitizer agents, chemotherapeutic drugs, small-molecule drugs, and therapeutic antibodies/vaccines, among others) (13). Chitosan-based NPs are shown to offer controlled drug release upon the inherent pH responsiveness of CS or in response to internal (expression of specific marker) or external stimulators (NIR) (14, 15).

### 2.4 Multifunctionalization capability

Due to the presence of functional groups, including hydroxyl and amine, Cs modification is possible through chemical cross-linking with other hydrophilic/hydrophobic polymers, such as polyethylene glycol (PEG), polysaccharides, sugars, short peptides, antibodies, and other functional groups (16). The purpose of functionalization varies from PEGylation to improve CS NP stability, ligand moiety for targeted drug delivery (17), labeling with imaging agents (18), synthesis of self-assembled vesicular NPS (19), affording stimuli-responsive sol-gel transition (20), generating different derivatives of CS (21), to advanced hybrid and composite versions. At the same time, CS can be modified with different functional moieties to afford theranostics (imaging and therapy) (8).

### 2.5 Targeted therapy

A specific derivative of chitosan named oligosaccharide chitosan (OCS) can act as an analog of hyaluronic acid (HA), with binding specificity to CD44 ligands. Accordingly, as a targeting moiety, NP decorated with OCS is developed for CD44^+^-overexpressing cells (22). Also, chitosan is shown to have an affinity for the MUC1 mucin adhesion molecule (CD227), a cell membrane glycoprotein, which is overexpressed in cancer cells such as glioma (23).

### 2.6 Transfecting agent: Gene delivery and endosomal escape

**Figure 1:**
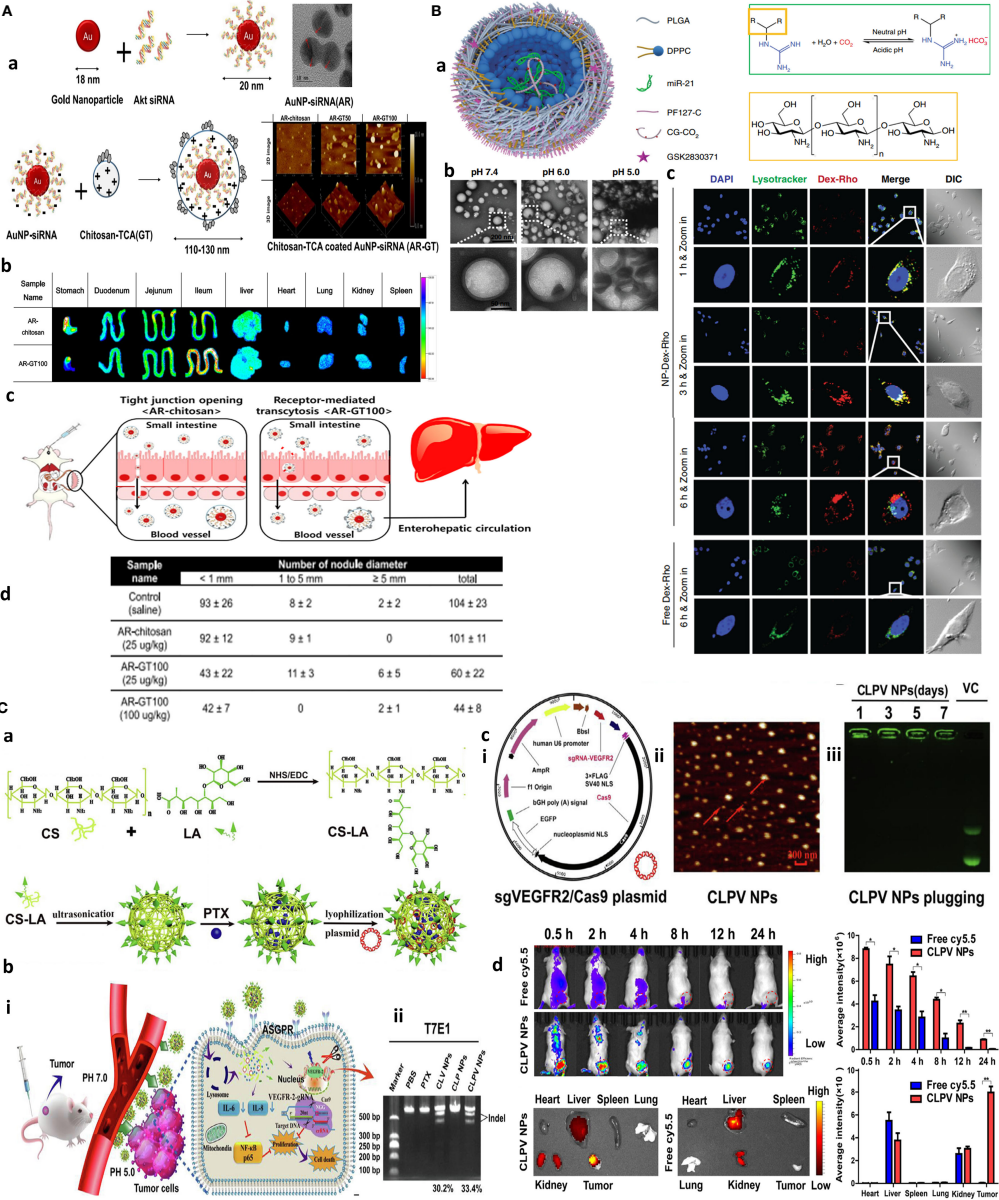
Chitosan NPs afford effective gene therapy and endo/lysosomal escape. **(A)** Bioinspired glycol chitosan NPs for GI passing and endosomal escape delivery of oral siRNA for successful treatment of CLM. **(a)** Synthesis and characterization of AR-GT NPs. **(b)** E*x vivo* fluorescence images of major organs of the AR-coated chitosan and glycol chitosan (GC)-coated NPs in treated animals for 30 min. **(c)** In vivo mechanism of AR-GT100 transport, which is designed to occur through transcytosis, inspired from enterocytic-mediated recycling of amphipathic fatty acid bilirubin, with specific accumulation in the ileum. For chitosan, this occurs through tight junctions. **(d)** Therapeutic efficacy in a CLM cancer mouse model after oral delivery of AR-chitosan and AR-GT100 (25 or 100 μg/kg) and CLM lung antimetastatic potential. Reprinted with permission from (24), Copyright (2017), American Chemical Society. **(B)** Chitosan NPs for precisely targeting POLR2A as a therapeutic strategy for human triple-negative breast cancer. **(a)** Scheme for NP synthesis. **(b)** TEM images showing pH-responsive NPs and nanobomb effect that occur under low pH 5.0–6.0 endolysosomes, leading to NP enlargement and cracking. At the same time, spherical core–shell NPs are intact under pH 7.4. **(c)** Endosomal escape of Dextran-Rhodamine (Dex-Rho) NPs. In early hours, the red color largely overlapped with LysoTracker green fluorescence. After 6 h, this overlap is minimum, indicating successful endosomal escape of core–shell chitosan-loaded anti-Mir21 due to the nanobomb effect. Reprinted from (25) Under Creative commons Attribution License 4.0, Copyright (2018) Springer Nature. **(C)** pH-responsive chitosan-based nanocomplex for efficient CRISPR/Cas9 gene-chemo synergistic HCC therapy. **(a)** Schematics of preparation and characterizations of CLPV NPs. **(b)** (i) Working principle of CLPV NPs for precise targeting of VEGFR2 and downstream tumorigenic pathways IL-6/IL-8-NF-κB p65; (ii) T7E1 analysis of the VEGFR2 sgRNA sites on tumor tissues. **(c)** (i) Plasmid profile of recombinant sgVEGFR2/Cas9 (VC); (ii) AFM image of 238.2-nm CLPV NPs; (iii) agarose gel electrophoresis analysis for VC stability on NPs. **(d)** *In vivo* biodistribution studies using injected free cy5.5 and cy5.5-loaded CLPV NPs recorded at different time points from the major organs and tumor tissues. Reprinted with permission from (26), Copyright (2018) Elsevier.

As the only positively charged polysaccharide due to the presence of amine groups in its structure, Cs DDSs possess several main advantages for gene therapy: (i) as to be complexed with negatively charged nucleic acids such as siRNA, shRNA, CRISPR-Cas9 mediated gene therapy, miRNA, CpG oligonucleotide adjuvants, and avoid their nuclease degradation; (ii) it acts as a transfecting agent, enabling cell-membrane adsorption and internalization of nucleic acid payloads; and (iii) it can withstand the proton sponge effect to escape the lysosome inside the target cells (tumor cells). Many advanced CS-based gene therapy approaches are designed to target and modulate a specific gene expression, silence oncogene gene expression, or upregulate a tumor suppressor gene expression (**Figure 1**). A review on chitosan-based gene delivery can be found in Ref (10).

Furthermore, transfecting ability and stability can be enhanced by the fabrication of CS-gold nanoparticles (AuNPs), as AuNPs are shown to be selectively internalized by tumor cells. Not to forget the effect of size and zeta potential in NP cellular uptake, the degree of PEGylation as a common NP stabilization and functionalization method to avoid “protein corona” formation can affect both size and charge of CS-based formulation as a transfecting agent. In this line, 5% PEG2000 Cs–AuNP displayed comparable transfecting potential to Lipofectamine 3000 with 75% gene knockdown. Using ELISA, the highest degree of protein c-MYC expression was achieved for Cs–AuNP with 2% PEG400 (~147 nm, −44.5 mV, 95%), >5% PEG400 (~154 nm, −33 mV, 90.5%), Cs–AuNP (~144 nm, −35 mV, 86.5%), 2% PEG2000 (~164 nm, −25.8 mV, 79.7%), and 5% PEG2000 (~171 nm, −25.5 mV, 75%) in a breast cancer cell model. This observation that PEGylation positively improved uptake of gold-CS nano complexes was irrespective of NP size and enabled efficient siRNA delivery compared to non-pegylated control (27). Among CS derivatives, N-acylated chitosan and chitosan oligosaccharide (CSO) are tested as plasmid DNA carriers complexed with AuNPs in cell culture (HEK-293). The transfection efficacy was validated by measurement of β-galactosidase activity and green fluorescence protein expression recorded at 27%, 33%, and 60% for chitosan, acylated chitosan, and CSO, with a TEM size of ~3.4, 4.6, and 7.3–15.6 nm, respectively, and positively charged zeta between 40 and 55 mV before complexation with plasmid DNA (28).

Other factors impacting siRNA delivery and its biodistribution involve chitosan molecular weight (~10 kDa for stability and knockdown), deacetylation degree and amine-to-phosphate (N:P) ratio, and increased surface charge (29). Taking advantage of pH-activated endolysosomal escape and drug release potential of chitosan, precise targeting of POLR2A using RNA interference (RNAi) is adopted as a therapeutic strategy for augmented cytosolic delivery of POLR2A siRNA for the treatment of human triple-negative breast cancer (TNBC) (30).

In a dual delivery system, folate-Cs-PEG nanoparticles were employed to encapsulate pRNA dimers, which simultaneously carry siRNA and aptamer, to selectively deliver it near or into target cells *via* aptamer-mediated endocytosis or proper particle size. Higher accumulation of siRNA in the tumor site, stronger tumor inhibition, and longer circulating time were also observed with CNPPs compared to other formulations *in vitro* and *in vivo*. Furthermore, pRNA hexamers can be designed to simultaneously carry six different substances (31).

The oral route is ideal for drug delivery as it is simple and allows multiple dosing if its bioavailability is not affected by gastrointestinal (GI) environments and nonspecific biological trafficking. Inspired by the enterohepatic recycling of bile acids, taurocholic acid (TCA) was coated on the surface of oral NPs to quarantine siRNA protection from GI degradation and facilitate colorectal liver metastases (CLM) targeting through the enterohepatic recycling process. An orally redox-responsive AuNP-thiolated siRNA-glycol Cs-taurocholic acid nanoparticle (AR-GT NPs) was developed in which Akt2 siRNA composed of dual padlocked nonviral vectors is conjugated with AuNPs and then covered by bifunctional glycol Cs-taurocholic acid through a charge-to-charge interaction. Three NPs were produced with different 50 and 100 AR : GT ratio complexation as AR-chitosan, AR-GT50, and AR-GT100 with mean diameters of 59 ± 10, 100 ± 3, and 115 ± 2 nm, respectively, with zeta potential values of AR-GT50 and ARGT100 of 29 ± 1 and 4 ± 1 mV. Among these, AR-GT100 was the most promising platform. It could selectively deliver siRNA to suppress AKT signaling, facilitate active transport through enterocytes, and enhance selective accumulation and inhibition of colorectal liver metastases (**Figure 1A**) (24). Another interesting study targeted 17q23 amplicon using a systemically delivered RNA-laden chitosan-based NPs capable of codelivery of GSK2830371 (WIP1 inhibitor), and antagomiR21 is reported to overcome the resistance to anti-HER2 therapy in the HER2+ breast cancer model. The core–shell NP with a diameter of 115 ± 9.7 nm was synthesized by a double-emulsion water-in-oil-in-water method, in which anti-miR21 inhibitor and pluronic F127 (PF127)-CG-CO2 were encapsulated in the inner water phase making an NP core. The shell structure contained hydrophobic GSK2830371 together with poly(d,l-lactide-co-glycolide) (PLGA) and 1,2-dipalmitoyl-sn-glycerol-3-phosphocholine (DPPC) dissolved in the oil phase. CS modification with the guanidine group (CG-CO_2_) formed chitosan-guanidine (CG) that reversibly reacts with carbon to produce carbon dioxide gas affording the nanobomb effect. More specifically, endocytosis-mediated uptake of the nanoparticles could generate CO_2_ to break open endo/lysosomes. This formulation protected antagomiR21 serum nuclease degradation up to 36 h and resulted in more than 95% tumor growth inhibition with significant efficacy on Her-resistant primary and metastatic tumors (**Figure 1B**) (25).

The clustered regularly interspaced short palindromic repeat (CRISPR)-associated Cas9 nuclease system (CRISPR/Cas9) has become a powerful tool for genome editing and gene knockout with better targeting effect than siRNA; however, its targeted delivery is challenging. To this, a β-galactose-carrying lactobionic acid (LA) functionalized pH-responsive chitosan-based nanocomplex is designed for combined gene chemotherapy by codelivery of sgVEGFR2/Cas9 plasmid and paclitaxel for hepatocellular carcinoma (HCC) therapy. LA was used to specifically deliver spherical CLPV NPs to asialoglycoprotein receptors (ASGPR) overexpressing HepG2 cells. Genome editing efficiency was 38.6% and 33.4% in difficult-to-transfect HepG2 cells *in vitro* and in tumor tissues *in vivo*, respectively, with more than 60% reduction in VEGFR2 protein expression and 70% tumor growth suppression in mice (**Figure 1C**) (26).

### 2.7 Immune-adjuvant

Chitosan deacetylation (DAc) of ≤17% and its high molecular weight and specific derivatives (*N*,*N*,*N*-trimethyl chitosan) are shown to elicit better immune-activating potential (32, 33).

Immune-adjuvants can increase vaccine efficacy by modeling the quality (type of immune response) and the host immune response quantity (rate and magnitude). Conventional adjuvants such as cytokines, toll-like receptor (TLR) agonist (CpG ODN), and aluminum salt stimulate the immune system. A good adjuvant, though, should be applicable for both systemic and local administration, safe, effective, low-cost, and capable of crossing physiological (mucosal/blood) barriers and immune targeting, all of which can be provided by chitosan (33). For example, a catalytic mucosal adjuvant strategy for an influenza WIV nasal vaccine based on CS functionalized iron oxide nanozyme (CS-IONzyme) is developed. CS modification not only increases antigen adhesion to nasal mucosa by 30-fold compared to H1N1 WIV alone; it also increased catalase-like activity of iron NPs for reactive oxygen species (ROS)-dependent dendritic cell (DC) maturation. This enhanced H1N1 WIV-loaded DCs migration into the draining lymph nodes for antigen presentation, resulting in 8.9-fold increase of IgA-mucosal adaptive immunity in mice and a 100% protection against influenza, compared to 30% protection by H1N1 WIV alone (34).

### 2.8 Mucoadhesion

**Figure 2:**
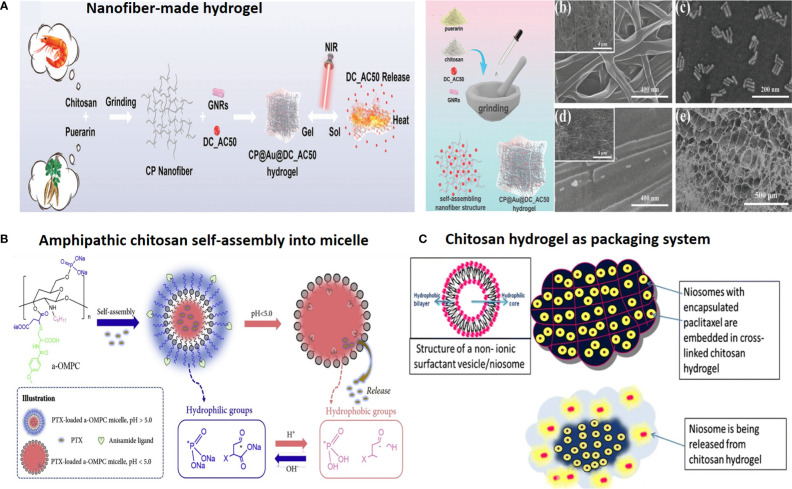
Chitosan-based drug delivery systems for cancer targeting. **(A)** Nanofiber-based chitosan hydrogel as injectable drug depot. Reprinted from (65) under Creative Commons Attribution License 4.0, Copyright (2021) Wiley‐VCH GmbH. **(B)** Amphipathic chitosan can self-assemble into vesicular core–shell particles. Reprinted with permission from (66), Copyright (2021) Elsevier. **(C)** Chitosan hydrogel as a packaging system for other NPs (niosome in hydrogel). Reprinted from (23) under Creative Commons Attribution License 4.0, Copyright (2020) Springer Nature.

Chitosan is shown to possess mucoadhesive properties, as it shows an affinity for the MUC1 adhesion molecule (CD227), a cell membrane glycoprotein, overexpressed by tumor cells. This property of chitosan is used for targeted oral delivery, as well as packaging and guiding of other small NPs, such as niosome-encapsulated paclitaxel conjugated with BODIPY 564/570. Accordingly, thermo-sensitive cross-linked chitosan hydrogel as a double package nanodelivery system was adopted for local, sustained, and timely burst release of niosomes upon targeting MUC1 mucin surface antigen overexpressing tumor cells (**Figure 2C**) (23). Also, chitosan-encapsulated niosomes are used for enhanced oral delivery of atorvastatin to hyperlipidemic mice. Chitosan encapsulation resulted in lower NP size, with 96.9 nm compared with 143.2 nm for unencapsulated niosomes (35).

### 2.9 Multitasking performance: Multimodal systems, theranostics, and combination therapy

Besides surface functionalization, chitosan NPs can afford simultaneous delivery of various payloads to form multifunctional NPs including theranostics (therapeutic and imaging agent) and multimodal or combination therapy employing more than one drug/strategy for a synergistic effect to overcome multidrug resistance (MDR).

#### 2.9.1 Multimodal/combination systems for reversing MDR

**Figure 3:**
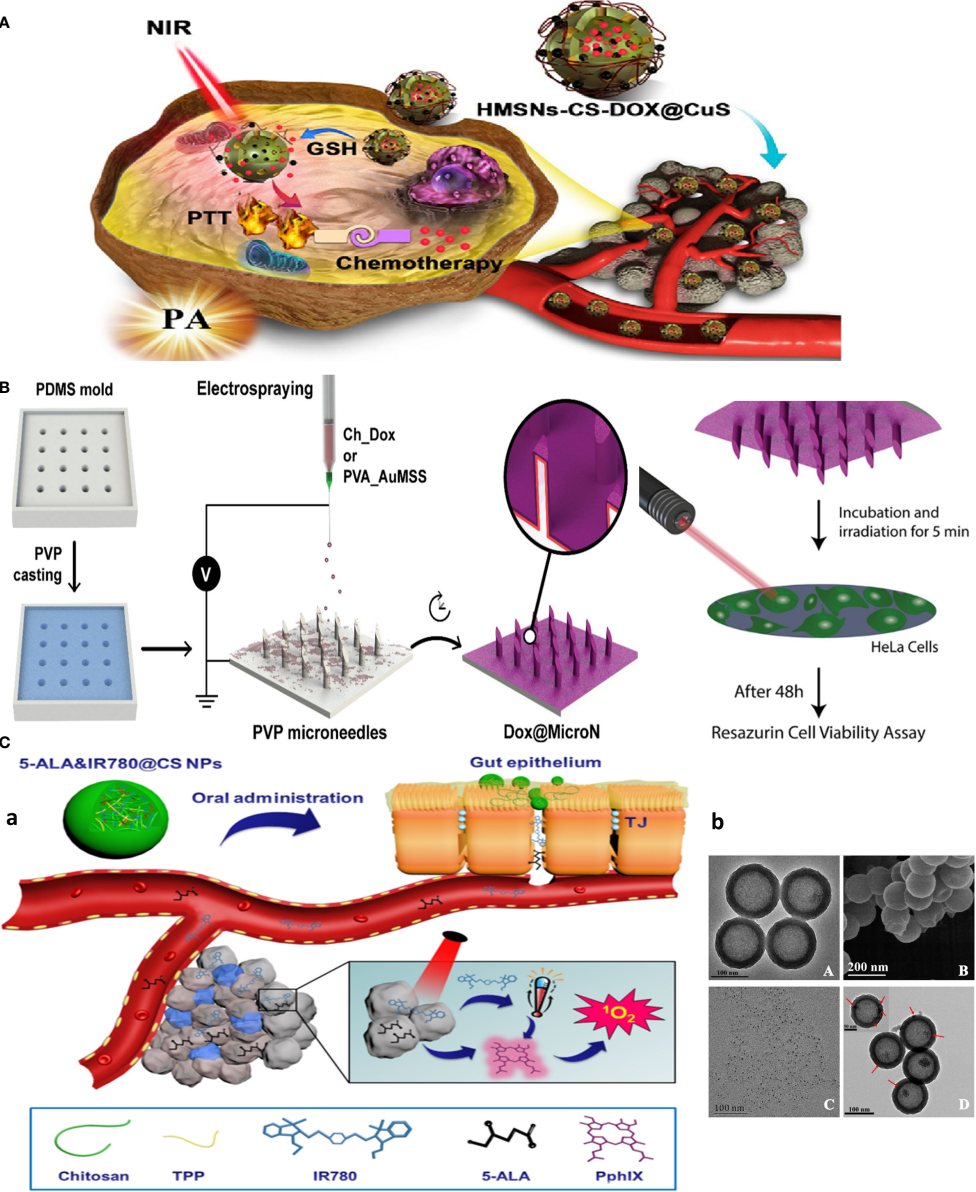
Chitosan-based multitasking drug delivery systems for treatment of MDR cancer. **(A)** Chitosan-based multimodal system for PTT-enhanced PDT therapy. Reprinted with permission from (36), Copyright (2020) Elsevier. **(B)** Chitosan MN patches for combined chemo-PTT cancer therapy. Reprinted with permission from (37), Copyright (2020) Elsevier. **(C)** Chitosan-based theranostics for thermal-photoacoustic (PA) imaging-guided tumor chemo-PTT therapy. **(a)** Schematic of NP working design. **(b)** Electron microscopy images: **(A)** TEM and **(B)** SEM images of HMSNs. TEM image of **(C)** CuS nanodots **(D)** and HMSNs-CS-DOX@CuS. Red arrows indicate the CuS nanodots on the HMSN surfaces. Reprinted with permission from (38), Copyright (2021) Elsevier.

As an example of multimodal system, CS NPs are used for encapsulating both photothermal (IR780) and photodynamic [5-aminolevulinic acid (5-ALA)] reagents for photothermally enhanced photodynamic therapy (5-ALA&IR780@CS NPs) for oral delivery and local treatment of CT-26 colon tumors in mice (**Figure 3A**) (36). Moreover, as a local delivery platform (tumor skin-inserted), microneedle (MN) array is attempted for cancer chemo- and photothermal combination therapy, in which micromolding and electrospraying techniques were used to produce layer-by-layer MN patches with polyvinylpyrrolidone (PVP) as the base of the needle. CS-DOX as an internal layer and polyvinyl alcohol (PVA) gold-silica nanorods (AuMSS) as external layers were deposited on the surface of the PVP MNs through the electrospraying procedure. MNs promoted the sequential delivery of doxorubicin and AuMSS in response to pH (chitosan) and photothermal stimuli (**Figure 3B**) (37).

Moreover, multifunctional chitosan NPs involving DOX-loaded chitosan-capped gold nanoparticles (CS-GNPs) are used for chemo-radiotherapy of MCF-7 breast cancer cells (39). Likewise, targeted cetuximab chitosan nanoparticles (Cet-CTS NPs) are used to deliver Quercetin (QUE) and paclitaxel (PTX) using an ionic cross-linking method. This formulation significantly decreased tumor growth in PTX-resistant xenografts (40). Given the highly promising results achieved with glycol chitosan (GC) for resistant cancer therapy, one study reported GC NP encapsulation of both chemotherapeutics (DOX) and siRNA (BCL-2) to achieve maximal efficacy to overcome MDR (41). Likewise, self-assembly of chitosan/vitamin E conjugate into micelle is used to entrap oxaliplatin (OXPt) to overcome conventional OXPt-mediated drug resistance/nephrotoxicity in breast cancer (42). Also, folic acid-functionalized chitosan nanocarrier is developed for codelivery of DOX and oleanolic acid (OA), to form a copolymer with self-assembly into 180-nm NPs in water. These pH-responsive FA-CS-g-OA@DOX NPs exhibit apoptosis-enhancing and antiproliferative capacities on MDR breast tumors *in vivo*, by sensitizing MDA-MB-231 cancer cells to DOX and mitigating DOX toxicity to healthy tissues (43). Furthermore, chemosensitizing NPs based on amphiphilic oligosaccharide CS-DOX–indomethacin conjugate are used for surmounting the efflux of cellular uptaken DOX *via* MDR-associated protein (MRP) in a lung tumor-xenografted mouse model (44).

#### 2.9.2 Theranostics (imaging-guided therapy)

Palladium, as a near-infrared plasmonic material alternative to gold NPs, was used for photo-based imaging and therapy owing to its near-infrared (NIR) heat generation potential for photothermal therapy (PTT) and generation of satisfactory photoacoustic signals for ultrasound imaging (45). The RGD peptide-functionalized oligosaccharide chitosan was coated on the surface of palladium nanoparticles (Pd@COS-RGD) to make biocompatible NPs acting as theranostics. Similar work adopted DOX-loaded hollow mesoporous silica nanoparticles (HMSNs) coated with chitosan-copper sulfide nanodots (HMSNs-CS-DOX@CuS), a theranostic, multimodal, and combination platform by merging chemotherapy and PTT into a single formulation. CuS dots are used to plug HMSN pores to avoid burst release of DOX into the systemic circulation, unless S–S bonds connecting the CuS dots to the HMSNs are selectively cleaved under the reducing microenvironment of the tumor, permitting targeted drug release. This system was capable of combinatorial chemotherapy and PTT, and multimodal thermal/photoacoustic imaging due to the incorporation of CuS nanodots for efficiently targeted tumor imaging and therapy (**Figure 3C**) (38). Also, self-assembly of amphipathic photosensitizer tetraphenyl chlorin (TPC)-chitosan (PS-CS) conjugate polymers into micellar nanoparticles in aqueous buffers are used for delivery of lipophilic drugs mertansine (MRT) or cabazitaxel (CBZ) for chemotherapy and PDT therapy of ferroptosis-resistant MCF-7 cells, with potential to be utilized as contrast agents (46).

## 3 Chitosan derivatives: Synthesis procedure

Despite the superior physicochemical as well as biological features of chitosan, the most significant drawback of this semi-synthetic biopolymer is the lack of solubility at pH values above its pKa (pH ~5.5−6.5), owing to the formation of interchain and intrachain hydrogen bonding between the hydroxyl and amine functionalities (16). The most important strategy for circumvention of this problem is chemical modification of chitosan through its hydroxyl or amine side groups. In this context, the popular methods used are *N*-substitution, *O*-substitution, *N*,*O*-substitution, chelation of metals, dendrimer attaching, fluorination, oxidation (e.g., with 2,2,6,6-tetramethyl-1-piperidinyloxy; TEMPO), depolymerization of chitosan to afford chitooligosaccharides (COS) *via* acid chitosanolysis, sulfonation, and polymer grafting (5). These modifications significantly improve its mechanical and thermal stability, acidic pH tolerance, and hydrophilicity, and can be used for controlling the interactions between chitosan and other elements, including drugs, metal ions, and organic compounds. The *N*- and *O*-substitution approaches are the most commonly used strategies toward chitosan derivatives (47).

**Figure 4:**
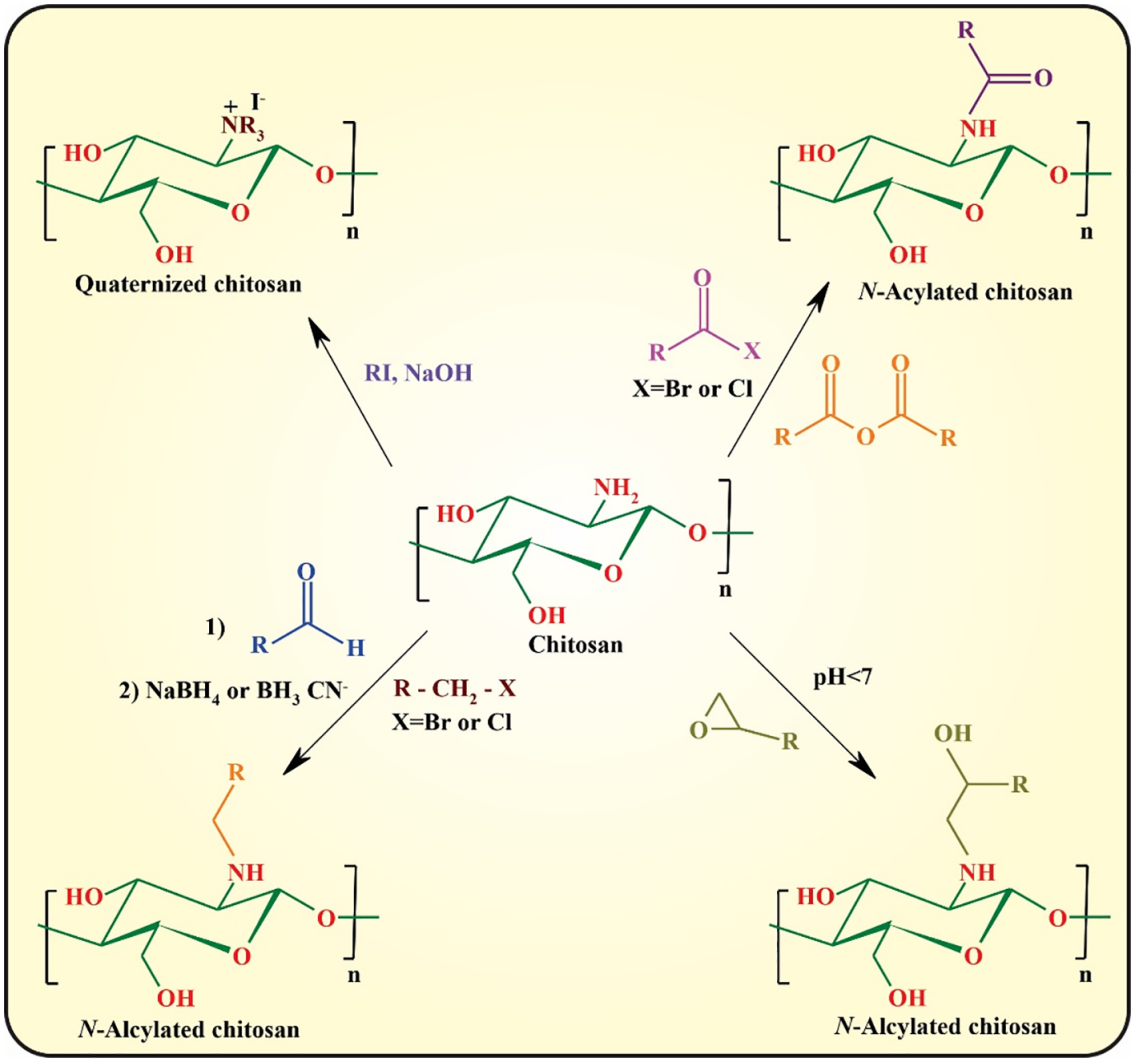
*N*-substitution of chitosan by different approaches.

In *N*-substitution, the amino group (-NH_2_) of chitosan reacts with various reactive reagents to afford chitosan derivatives. The most important approaches for the *N*-substitution of chitosan are quaternization, *N*-alkylation, and *N*-arylation with aldehydes through “Schiff-Base” reaction followed by the reduction of the formed imine group to the more stable amine group, epoxy, and reactive halide derivatives, and *N*-acylation with anhydride and acid halides (**Figure 4**) (45, 48).

**Figure 5:**
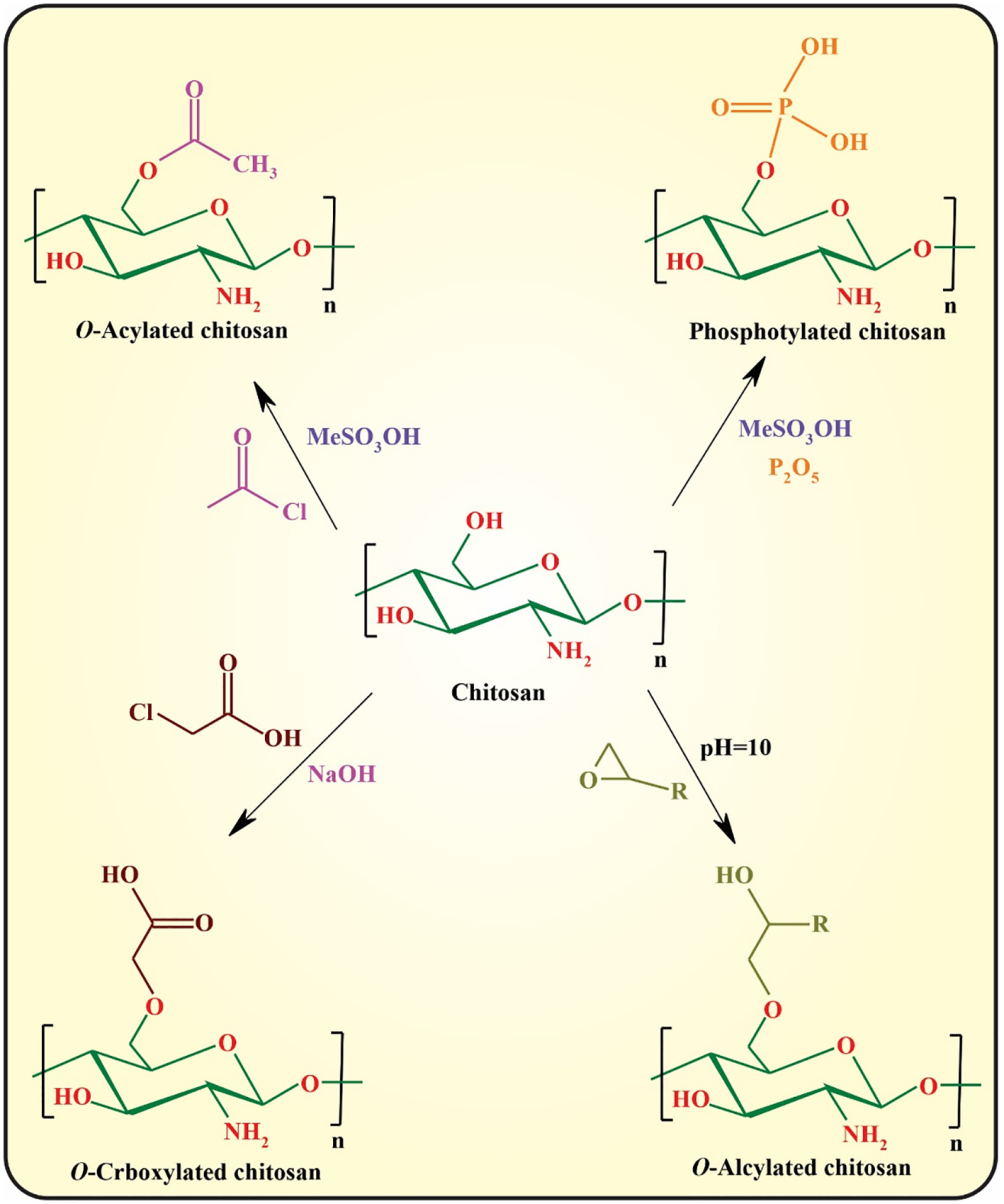
*N*-substitution of chitosan by different approaches.

*O*-substitution of chitosan is another efficient approach for improving the physicochemical properties of chitosan that regularly requires the protection/deprotection of its amino groups owing to the higher reactivity of amino groups than those of the hydroxyl groups. The most commonly used approaches toward *O*-substituted chitosan are illustrated in **Figure 5** (48, 49).

Amidation with coupling catalysts [e.g., *N*-(3-dimethylaminopropyl-*N*-ethylcar-bodiimide) hydrochloride (EDC)/*N*-hydroxysuccinimide (NHS)] is another important approach for functionalization of chitosan using its amino group. This approach can be used for fluorination as well as conjugation of bioactive small molecules or macromolecules (e.g., folic acid and RGD peptide) (50). Finally, polymer grafting can be considered an efficient strategy for modified chitosan with tunable physicochemical and biological properties.

This polymerization technique is one of the most exploited approaches toward chitosan graft copolymers. The grafting process can be achieved through ceric-initiated and Fenton’s free radical graft copolymerization that is used for vinyl monomer grafting, cyclic monomer grafting *via* ring-opening copolymerization, post-grafting *via* various coupling agents (e.g., click chemistry, EDC/NHS, and nucleophilic reactions), and reversible deactivation radical polymerization (RDRP; also famous as controlled or “living” radical polymerization) approach (51). The RDRP technique is categorized as atom transfer radical polymerization (ATRP), nitroxide-mediated polymerization (NMP), and reversible addition-fragmentation chain transfer (RAFT) polymerization that allows the synthesis of well-defined macromolecular architectures with relatively low dispersity (52–54).

## 4 Chitosan derivatives: Formats and features

Herein, we aim to overview different formats and applications of most important chitosan derivatives for cancer therapy.

### 4.1 Glycol chitosan: Targeting tumor heterogeneity and lymph node trafficking

GC is a chitosan derivative with much better solubility as it is conjugated with hydrophilic ethylene glycol branches. This modification renders GC soluble in neutral/acidic pH, while the amine groups of GC remain unaffected for further modification/functionalization. The molecular weight of GC (20 to 250 kDa) and the degree of deacetylation (60% to 82.7%) affect GC’s physicochemical and biological properties. Among Cs derivatives, GC is a well-known nanoparticle for cancer therapy, particularly for cancer heterogeneity (5). Moreover, cationic particles are known to promote trafficking and retention of therapeutic payloads in lymph nodes. For example, a dendrimer-based icluster nanobomb is shown to specifically inhibit lymph node metastasis, after size changes in the primary tumor site (∼100 nm to ∼5 nm) wherein resultant small NPs promoted particle intravasation into tumor lymphatics and migration into LNs, and complete tumor remission in 4 out of the 10 mice for 110 days (55). Meanwhile, GC-based theranostics, such as glycol-chitosan-coated gold nanoparticles (GC-AuNPs), are employed for a photoacoustic contrast agent imaging of lymph nodes without the need for targeting moiety/surface modification and are capable of tumor antigen delivery to macrophages to present the model ovalbumin (OVA) epitope at targeted lymph nodes for cancer immunotherapy (56).

### 4.2 Fluorinated chitosan: Mucoadhesion/forming stealth NPs

As an additional modification, fluorination of functional drug delivery system materials can result in satisfactory performances such as improved stability, resisting protein adsorption, and enhancing cell membrane permeability. In this regard, fluorinated chitosan is used for enhanced transmucosal delivery of different cargoes such as catalase or chemotherapy agents (57, 58). Fluorination can form stealth NPs to avoid protein corona, reduce RES capture, and improve drug accumulation in tumor sites (59).

### 4.3 Oligosaccharide chitosan: Anticancer and cell-specific targeting

CSOs, which are obtained from chitosan degradation, possess some interesting molecular weight-dependent biological properties, especially anticancer activity (60). CSO has a low molecular weight, is water-soluble, and acts as a specific HA analog. CSO-decorated NPs can be used for CD44-overexpressing cancer tumor cells, such as TNBC cells (22).

### 4.4 Carboxylated chitosan: Functionalization, pH responsiveness, drug release, and endo/lysosomal escape

Chitosan possesses a positive charge; however, sometimes a negative charge is desirable to achieve additional features. Such carboxylated chitosan is used to improve the efficacy of mesoporous silica nanoparticles for targeted drug delivery of HER2-overexpressing breast cancer. This involved chitosan-coated, DOX-loaded anti-EGFR/HER2 aptamer-mesoporous silica nanoparticle (MSN) bioconjugates. Partially carboxylated chitosan as coating imparted pH responsiveness and greater DOX release in a shorter time, endo/lysosomal escape ability to MSN delivery for cytosolic delivery of DOX, and conjugation targeting agents (aptamer) for active targeting (61). Also, genistein (C_15_H_10_O_5_), a soy isoflavone and superparamagnetic Fe_3_O_4_ decorated with bio-compatible carboxymethylated chitosan, is used to target acute leukemia lymphoma (62).

### 4.5 Quaternized: Antimicrobial and tissue regeneration

Though the antimicrobial activity of chitosan is affected by pH, molecular weight, and the presence of interfering substances like lipids and proteins, CS modification such as succinoylated, hydrophobic, sulfonated, sulfonamidated, and in particular quaternary can enhance its antimicrobial performance (30). Quaternized chitosan (QCS), is a water-soluble chitosan derivative with good biocompatibility and enhanced antibacterial effects. Due to the presence of positively charged cations in its amino and quaternary amine groups, QCS possesses strong tissue adhesive properties and can induce tissue repair, hemostasis, and regeneration. In addition, quaternized aminated chitosan (Q-AmCs) derivative is used for the efficient encapsulation and slow release of the therapeutic drugs (21).

### 4.6 Cathecholic CS: Wet adhesion and hemostatic

Inspired by the super adhesion performance of mussel to rocks owing to the specific amino acid content of its adhesive proteins Mfp-3 and Mfp-5, namely, catecholic amino acid (3,4-dihy-droxyphenylalanine, DOPA) and L-lysine (63), catechol groups are used in the fabrication of Cs possessing super adhesion potential under wet conditions, such as surgical trauma and internal bleeding.

### 4.7 Sulfated: Angiogenesis inhibitor

Chitosan sulfate inhibits angiogenesis *via* blocking the VEGF/VEGFR2 pathway and suppresses tumor growth *in vivo*. Heparinoid chitosan sulfate inhibits neovascularization much higher than heparin control (63.8% *vs*. 30.7%) and possesses anticoagulant activity *in vivo* (64).

### 4.8 Amphiphilic: Self-assembled NPs

Chitosan-based drug delivery systems can be prepared using different methods such as chemical or ionic gelation, mini-emulsion, spray-drying, and coacervation/precipitation methods. An alternative to these is self-assembly, a spontaneous process for easy making of organized structures with particular functions, achieved by amphiphilic chitosan derivates (19).

For example, anisamide-conjugated *N*-octyl-*N*,*O*-maleoyl-*O*-phosphoryl chitosan (a-OMPC) amphiphilic micelles featuring pH-responsive release and high affinity to sigma-1 receptor-overexpressed tumors are developed for paclitaxel (PTX) delivery. Maleoyl functionalization of chitosan has several advantages: (i) improved chitosan solubility in organic system, (ii) endo/lysosomal pH-responsive drug release due to conversion from phosphate/carboxylate to hydrophobic phosphoric acid/carboxylic acid, (iii) and providing α,β-unsaturated ketone double bond for anisamide conjugation, to afford chitosan a high affinity to bind sigma-1 receptor overexpressed on the surface of prostate cancer (PCa) cells (**Figure 2B**) (66).

Pluronic is an amphiphilic tri-block copolymer composed of poly(ethylene oxide) (PEO) and poly(propylene oxide) (PPO) and can form micelles for delivery of both hydrophobic and hydrophilic drugs within the hydrophilic PEO and hydrophobic PPO blocks forming the corona and the core of the micelles, respectively. To overcome the instability of polymeric micelles made of pluronic^®^F127 (PF), the pluronic-chitosan copolymer is exploited. Aptamer-modified pluronic^®^F127 and chitosan are used to enhance PTX loading capacity and increase micelle stability (86.22 ± 1.45 nm) (67). Likewise, owing to the temperature-responsive potential of pluronic, the poloxamer 407-chitosan copolymer is used to develop thermoresponsive hydrogels as drug depots and single-shot vaccines capable of synchronous and sustained release of lactic-co-glycolic acid (PLGA) nanoparticles loaded with antigen and adjuvants. This chitosan hydrogel vaccine induced strong, long-lasting, humoral cellular responses in the murine melanoma model (68).

## 5 Types of chitosan nanoparticles

Chitosan and its derivatives can be formulated into different types of NDDSs; the most popular forms include hydrogel, vesicles, nanoparticles, and composite/hybrid ones (**Figure 2**).

Through ionic or chemical gelation, CSO can form 3D cross-linked/entangled hydrophilic networks, known as hydrogels. Under their LCST, hydrogel can adopt state transition from gelation (gel) to solution (sol) and *vice versa*. Another classification is based on the hydrogel route of administration, to be used as drug depot (injectable/sprayed hydrogel), systemic delivery (nanogel with size <200 nm), or microgel (bulk gel), which comes in different forms such as fiber, cryogel, sponge, film, patch, and glue with the application as wound dressing or scaffold. The sol-gel transition capability is important for the formulation of injectable hydrogels, upon injection into the tissue site, where gelation and local entrapping of cargo occur. Thus, it serves as a drug depot for long-lasting release. Chitosan can also be cross-linked with hydrophobic moieties, to form amphipathic copolymers that can self-assemble into vesicular particles such as micelles and liposomes. Also, CS NPs alone or complexed with other materials/NPs (e.g., chitosan-dendrimer) and polysaccharides (e.g., chitosan-alginate) form composite or hybrid NPs. This capability further extends and advances CS application into a single all-in-one robust platform with multitasking potential for imaging, therapeutic, and prevention of recurrent disease in a controlled manner.

## 6 Tumor-penetrating chitosan NPs cross biological and microenvironmental barriers

Considering the solid TME, various physiological barriers exist in the way CS NPs advance toward the tumor parenchyma. In this section, we aim to introduce these critical barriers and formats of CS-based drug delivery systems that take advantage of CS inherent properties to build effective therapeutics to fight recurrent cancer and MRD.

### 6.1 Blood barrier

The first barrier for systemically injected CS NPs is the formation of a protein corona, which results in the clearance of NP by the phagocytic system and diminished circulation time of NP. This barrier can be tackled by using protein corona repelling approaches, using dysponins such as PEGylation, BSA coating, protein corona formation, and cell-derived camouflaged membranes. Concerning CS, mucoadhesive properties and its cationic charge act as a double sword: it affords adsorption-mediated cross-linking through cell membrane; in the same way, it could also possess high fouling properties to bind with serum-abundant proteins such as albumin, complement proteins, immunoglobulins, and, thus, phagocytosis (69). Meanwhile, as discussed before, the fluorinated form of CS can possess repelling properties and act as stealth coating (59). A recent paper detailed these strategies implemented to increase the stability of systemically administered chitosan nanoparticles. Besides PEG, different coating materials, using polysaccharides, chitosan derivatives, stealth chitosan, and zwitterionic chitosan plus their advantages and disadvantages are also discussed (69).

### 6.2 Membrane/stroma barriers

After the protein corona is tackled, NP must cross biological barriers to penetrate tumor cells. These include crossing the vascular wall (intravasation), then dense ECM and stroma cells (convection) and finally tumor cell membrane (internalization). The first one, vascular wall, is for systemically injected NPs, while the rest are common between locally and systemically injected NPs, as for CS-based DDSs. These barriers can be targeted using active/passive targeting, multi-stage acting NPs, nanosweepers, and Trojan systems discussed in the next section.

#### 6.2.1 Ligand and ligand-independent delivery

Unlike what has been accepted for a long time, that enhanced permeation and retention (EPR) is the main inactive route for targeted delivery of NPs to solid tumors, recent meta-analysis data contradicted this fact as it indicated that only a mean of 1.48% of the injected dose (%ID) of NPs is delivered to cancer cells (70). Vascular permeability determines NP localization in tumor lesions. A higher NP accumulation, besides EPR, needs an active targeting by using ligand-decorated NPs to enjoy the transcytosis mechanism to promote both intravasation and internalization of NPs.

Different ligands can be used to achieve this; however, a ligand of choice is the one that is expressed abundantly by both tumor and tumor-associated endothelial cells, enabling a DDSs to tackle several barriers using a single targeting moiety, here, vascular wall and tumor cell membrane internalization (71). Examples include NGR, which targets CD13 expressed by both tumor and endothelial cells (72). Likewise, the cell-penetrating peptide iRGD containing the CendR sequence can first target integrin receptor to cross the blood–brain barrier and then provides the second moiety to bind Neuropilin-1 (NRP-1) and Neuropilin-2 (NRP-2) for crossing the brain–tumor barrier (73). Lactoferrin receptor (LfR) is another example with dual expression on tumor and endothelial cells for glioma therapy (71). Chitosan micelles incorporating Telmisartan can sequentially target overexpressed angiotensin II type I receptor (AT_1_R) on CAFs and tumor cells (74). Bispecific antibodies can also provide dual-targeting ability of NPs using a single formulation (75). Other approaches employ a single receptor either for tumor cell or endothelial cells targeting combined with stimuli responsiveness behavior (multi-stage NPs), or polyplexes/cationic particles [such as chitosan, dendrimer, and polyethyleneimine (PEI)], cationic cell-penetrating peptides (CPP), and cationic lipids to afford adsorption-mediated internalization.

As both OCS and HA show affinity to selectively bind CD44-expressing cells, self-cross-linkable chitosan-HA dialdehyde nanoparticles are used for CD44-targeted siRNA delivery to target oncogene Bcl2 for bladder cancer therapy (76). Likewise, the HA-CS nanocomposite can be used for the generation of polyionic nano-complexes, which affords CD44-mediated active delivery of both positively charged (DOX) and negatively charged (mir-34-a) cargoes for the treatment of MDA-MB-231 breast cancer cells (77).

Besides affording ligand-mediated endocytosis, cyclic RGD (cRGD) can possess antiangiogenic and antimetastatic potential by inhibiting ανβ3 integrin receptors (78). For one, cRGD-decorated chitosan NPs are used for encapsulating chemotherapeutic drug raloxifene, while free amines of chitosan afforded NP stability under low pH of TME and realized active targeted delivery to selectively suppress angiogenesis and tumor growth in breast cancer (79).

**Figure 6:**
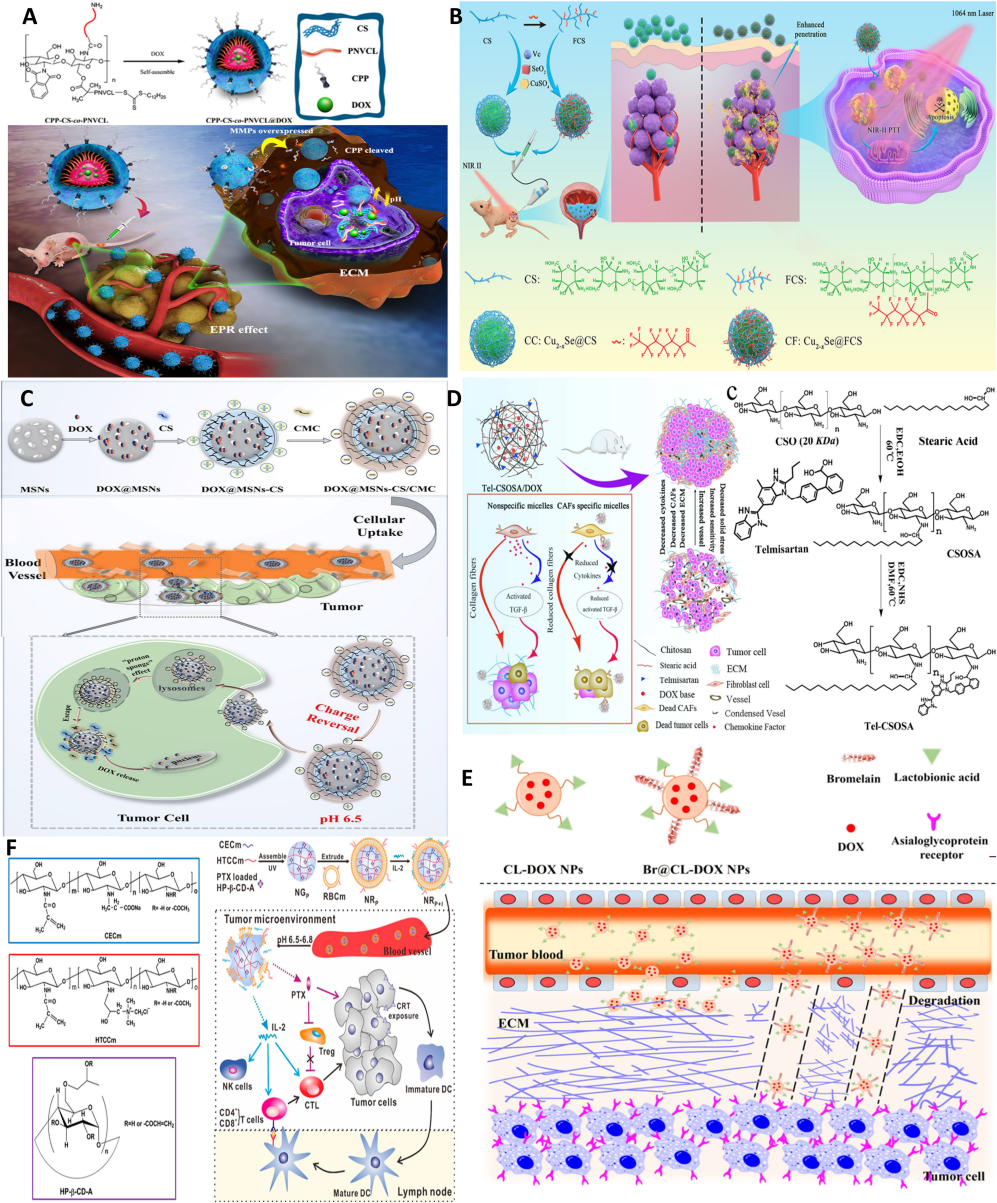
Chitosan NPs affords crossing stroma and biological membranes. **(A)** EPR and CPP-mediated active targeting. Reprinted from (15) under Creative Commons Attribution License 4.0, Copyright (2019) Springer Nature. **(B)** FC affords superior mucoadhesion potential to cross epithelial barriers of bladder cancer. Reprinted with permission from (80), Copyright (2021) Wiley‐VCH GmbH. **(C)** Multi-stage acting CS NPs. Reprinted with permission from (81), Copyright (2019) American Chemical Society. **(D)** Stroma cell depletion afforded by chitosan-modified CAF inhibitor. Reprinted with permission from (74), Copyright (2018) Elsevier. **(E)** Chitosan nanosweeper for ECM drilling. Reprinted with permission from (82), Copyright (2018) Elsevier. **(F)** Chitosan-clocked NPs with RBC membrane as Trojan system. Reprinted from (83), Copyright (2017) American Chemical Society.

In another formulation, targeted delivery is afforded using ligand-based delivery coupled with microenvironment-responsive properties. A chitosan-based cascade−responsive nanoplatform is developed for TNBC therapy involving dual pH/thermosensitive poly(N-vinylcaprolactam) (PNVCL)-CS copolymer modified with the CPP (CPP‐CS‐co‐PNVCL NPs) and loaded with DOX. Core–shell self-assembly of amphipathic 200-nm NPs with DOX entrapped in the hydrophobic core occurs due to amphiphilic block copolymers. Upon systemic injection, NPs are intact while reaching the tumor site, and the bond between CPP and CS is degraded by matrix metalloproteinase enzymes. *In vivo* experiments employing a TNB xenograft mouse model demonstrated a significant reduction in tumor volume and life span, with no obvious systemic toxicity (**Figure 6A**) (15).

On the other hand, as discussed earlier, Cs, particularly its fluorinated derivative (FC), can be used for enhanced mucoadhesion of NPs and deep tumor cell penetration by coupling FC to sonosensitizer-conjugated catalase post-intravesical instillation. FC but not CS can open tight bladder epithelium junctions by reversible modulating transepithelial electrical resistance (TEER) (57). Likewise, FC is coupled to copper selenide nanoparticles for NIR-triggered (**Figure 6B**) (80) as well as photo-activated H2 generation using a catalyst [FeFe]TPP for hydrogen-chemotherapy (gemcitabin) of bladder cancer (58).

#### 6.2.2 Charge/size-shifting NPs

As discussed before, chitosan and its derivatives, as a cationic biomaterial with mucoadhesive potential, afford a ligand-independent yet active delivery platform to cross and transport across cellular membranes and successive endocytosis. Nonetheless, positive charge is a problem for protein corona formation, and thus, a microenvironment-responsive multi-stage acting NPs are desirable with the capacity for charge/size-shifting. For example, surface-charge-switchable chitosan nanoclusters (CMGCC) as synergistic theranostic are fabricated for T1-weighted magnetic resonance imaging and intracellular antioxidant glutathione (GSH) depletion-enhanced PDT. CMGCC contains chlorin-e6-conjugated GC polymeric micelles incorporating catalase-stabilized MnO_2_ nanoparticles. In this system, GC polymers with pH-sensitive surface charge switchability from neutral (pH 7.4) to positive (pH 6.5) could improve the PS accumulation within the tumor region. At the same time, the combined work of CAT and MnO_2_ could effectively consume H_2_O_2_ and GSH to reoxygenate the hypoxic tumor *via* catalyzing endogenous hydrogen peroxide to O_2_, and ^1^O_2_, respectively; meanwhile generated Mn^2+^ serves as a contrast agent. Thus, a charge-reversal activity that only occurs under the low pH of TME restores the positive charge of GC for PS accumulation and subsequent enhanced PDT efficacy toward subcutaneous Hela tumors (84).

In another preparation, core−shell hybrid MSNs-CS/CMC NPs composed of mesoporous silica nanoparticles (MSNs) stabilized by positively charged OCS and negatively charged carboxymethyl chitosan with pH-triggered charge-reversal potential are used for effective intracellular delivery of DOX. The pH-triggered charge-reversal CS/CMC bilayer was key to NP internalization, endosomal escape, and DOX release. The surface charge of DOX@MSNs-CS/CMC was reversed from −16 (pH 7.4 in blood) to 11 mV (pH 6.5 in TME) and 15 mV (pH 5.5 in endosome). Thus, the reversible protonation–deprotonation strategy using CS derivatives provides an alternative ligand-independent platform for efficient drug delivery in breast cancer therapy (**Figure 6C**) (81).

Another form of multi-stage acting NPs is those that use an ECM-biodegradable coating such as gelatin to be degraded by matrix metalloprotease or hyaluronidase two abundantly expressed enzymes in TME. This strategy is specifically useful as the transport of polycationic NPs such as chitosan can be hindered due to the presence HA, which tends to form cationic/anionic complexes. Thus, HA coating protects the cationic core, and after degradation, in the second stage, the positive charge of the core element, e.g., chitosan, can be revealed to promote cellular uptake. Detailed examples of change-shifting NPs as a category of multi-stage acting NPs are reviewed in Refs (85, 86).

#### 6.2.3 Nanosweepers

Dense stroma and ECM as seen for fibrotic/desmoplastic tumors such as breast, HCC, and pancreatic ductal adenocarcinoma (PDAC) can dramatically diminish NP convection. They may result in heterogeneous accumulation of NPs within solid tumors. Thus, strategies are required to afford deep NP penetration by ECM normalization or stroma cell targeting. The main targets are collagen, HA, and cancer-associated fibroblasts (CAFs) as the main constituents and contributors to the formation of dense ECM in desmoplastic tumors (87).

As one strategy, ECM-degrading enzymes, such as collagenase, can be decorated on the surface of NPs, acting as sweepers, as they digest ECM components along their way. Collagen I depletion is another strategy reported by inhibitors of collagen synthesis, such as lysyl oxidase or collagen-degrading enzymes, e.g., collagenase (88). Also, losartan is shown to improve oxygen distribution in tumor tissues by collagen depletion, allowing for deep percolation of paclitaxel-encapsulated liposomes (89). MSN conjugated with pineapple-derived peptidase papain family, bromelain (Br), can drill through ECM and enable the uptake of the particles by endothelial, macrophage, and cancer cell lines (90). Such lactobionic acid-modified bromelain-immobilized chitosan nanoparticles are used for enhanced active targeting and drug penetration respectively in tumor tissues (**Figure 6E**) (82).

ECM loosening is attempted by reprogramming stroma cells involved in collagen synthesis, particularly CAFs. Several strategies such as ultrasound (US) treatment, sonodynamic therapy (SDT) with tens of centimeters of depth penetration in soft tissue (57), hyperbaric oxygen, or PTT can also afford deep tumor penetration by modulating tumor permeability and ECM remodeling. In this line, deep tissue penetration of nanoparticles using US treatment improves EPR-based passive targeting efficiency of nanoparticles *in vitro* and *in vivo*. For one, pulsed high-intensity focused ultrasound (pHIFU) NPs are utilized to temporally improve blood perfusion, extravasation, and tissue penetration of fluorescent dye-labeled GC nanoparticles (FCNPs) in femoral tissue of mice by 5.7-, 8-, and 9.3-fold compared to that of non-treated ones (0 W pHIFU) (91). Hyperbaric oxygen therapy is also used for reversing hypoxia and elimination of CSCs by modulating depleted excessive ECM, such as collagen and fibronectin, and thus normalized tumor blood vessels both structurally and functionally and was effective in enhancing the efficacy of commercialized nanomedicines, Doxil and Abraxane, against TNBC and PDAC as stroma-rich solid tumors (92). As an example of dual-targeting ligands, chitosan micelles are formulated, which can target both tumor and most abundant stroma cells, CAFs, that localize near blood vessels within “finger-like” collagen-rich stroma, and lead to restrained drug transport. As mentioned before, biologically inspired Telmisartan grafting glycolipid micelles (Tel-CSOSA) can specifically eliminate CAFs and tumor cells. Uniform and deeper penetration of injected Tel-CSOSA/DOX NPs was achievable due to CAF apoptosis, which eliminates “finger-like” ECM, enforces subsequent tumor cell targeting, and renders tumor cells more vulnerable to chemotherapeutics (**Figure 6D**) (74).

#### 6.2.4 Trojan system

An additional strategy to help transport DDSs across biological membranes and stroma is biomimetics inspired by the ancient “Trojan horse”, which aims to smuggle cargoes as close to the tumor nucleus as possible. This can be achieved using bacteria, macrophages, and stem cells that show inherent tumor homing tropism toward a hypoxic microenvironment through chemokine gradient, or using biomimetic/camouflaged NPs that adopt cell membranes of other types of cells including immune cells, tumor cells, red blood cells, platelets, exosomes (86), and MSCs. This way, NP coated or encapsulated within cells/cell membranes cannot be recognized by immune cells in the circulation; meanwhile, deep intratumor penetration can be afforded through hypoxia/chemotaxis gradients that drive trafficking of Trojan NPs towards solid tumors.

Chitosan, as a cationic polysaccharide, can bind with the glycoprotein receptors such as mannose receptors, Dectin 1 receptors, and TLR2 and TLR4 expressed in blood cells, monocytes, macrophages, and DCs. This, in turn, results in the endocytic uptake of chitosan-based DDSs by these cells. During recruitment to a diseased site, differentiation of monocytes to macrophage occurs. With this in mind, chitosan NP packaging in the blood is attempted by Ly-6C^hi^ monocytes, which took up COSA micelles. Furthermore, the micelles in macrophages can be exocytosed and subsequently taken up by cancer cells (93).

Also, in the light of antitumor activity of tumor necrosis factor α (TNF-α) and given the fact that macrophages can produce TNF-α in response to lipopolysaccharide (LPS), time-controlled expression of TNF as a transmembrane-expressed coating was induced to produce macrophage-tethered TNF-α to coat chitosan NPs. This biodegradable biomimetic module was effective against MCF-7, Hela, and MDA spheroids *in vitro* (94). Finally, RBC membrane-coated pH-responsive chitosan nanogel is used for combinatorial cancer chemoimmunotherapy. Nanogels were formulated with hydroxypropyl-β-cyclodextrin acrylate for paclitaxel loading and two opposite charged chitosan derivatives, namely, amphoteric methacrylamide N-carboxyethyl chitosan (CECm) and positively charged methacrylamide N-(2-hydroxy)propyl-3-trimethylammonium chitosan chloride (HTCCm), to precisely control the pH-responsive capability. Furthermore, biomimetic coating with erythrocyte membrane achieved “nanosponge” property and enhanced adsorption (through an IL-2-like agonist glycoprotein on RBC membrane), protection, and delivery of IL-2. Upon localization in tumor site by EPR, nanogel swelling exposed PTX-loaded HP-β-CD-A for low-dose sustained release of PTX, and induced calreticulin (CRT) exposure on tumor cells, along with DC stimulation and maturation, which finally activated immunosurveillance. After losing the inner core support, the membrane could be disintegrated to constantly release IL-2 into the TME for stimulating cytotoxic T lymphocytes (CTLs) and natural killer (NK) cells (**Figure 6F**) (83).

### 6.3 Microenvironmental barriers

#### 6.3.1 Hypoxia (deep-seated tumor cells)

As a solid tumor grows up to a few millimeters in volume, its demand for oxygen and nutrients relies on neovascularization to provide blood supply. However, the high metabolic activity of tumor cells forces them to adopt a chaotic, unstructured, malfunctioning vascular system and anaerobic glycolysis (95). As a result, the TME is acidic, and there exists a gradient and heterogeneous concentration of oxygen levels within different parts of tumor layers, with a hypoxic/necrotic core and well-perfused proliferative cells at the tumor periphery (4). Hypoxic cells are characterized by upregulated expression of hypoxia-inducible factor (HIF-1a) residing in the deep regions within the tumor, rendering them inaccessible to therapeutic drugs. Also, heterogeneity concerning different pH values, oxygen, ATP, enzymes, tumor-specific receptor expression, etc., results in the uneven distribution of drugs/nanoparticles and, thus, their suboptimal efficacy towards cancer cells (3, 96). Among TME hallmarks, the role of hypoxia in cancer evolution and adaptation is well regarded. Hypoxia promotes ROS-induced genomic instability, alters DNA repair pathways, inhibits apoptosis, and promotes autophagy, tumor metabolism, angiogenesis, and the emergence of CSCs through promoting epithelial–mesenchymal transition (EMT) and immunosuppression. More importantly, a vicious cycle of hypoxia is responsible for the selection of new and more invasive and resistant clones that fail the efficacy of currently practicing anticancer regimes, resulting in recurrent cancer (97).

There are three general anticancer strategies based on tumor hypoxia: (i) harnessing it for the development of bioresponsive (hypoxia-responsive) nanomedicines; (ii) alleviating the hypoxia using vascular normalization agents (antiangiogenic therapy) or increasing oxygen level externally [oxygen delivery using hemoglobin (98) or perfluorocarbon] or internally [using oxygen generators called enzymes, which employ MnO_2_ (99) and catalase (CAT) (100) to generate O_2_ from H_2_O_2_ present abundantly in TME]; and, finally, (iii) oxygen depletion or starvation therapy, which aims to kill tumor cells by exacerbation of hypoxia and oxygen/nutrient depletion, as practiced by vascular infarction/disruption therapy using vascular disrupting agents or delivery of thrombosis-inducing agents such as coagulase fusion proteins (101, 102) or thrombin delivery (103) using a nanorobot to the tumor site to induce thrombosis selectively in tumor blood vessels. Oxygen depletion itself can be achieved externally by PDT and radiotherapy, which consumes intratumoral O_2_ to generate ROS or endogenously by inherent oxygen-consuming materials such as magnesium silicide (Mg_2_Si) (104), perfluorotributylamine (PFTBA) (105), or glucose oxidase (GO_X_) (106). Mostly, for a better efficacy, the combination of these approaches together (107) or with other approaches such as chemotherapy (99), radiotherapy, SDT (100), immunotherapy, and PDT (108) is desired. This can be well-achieved using CS as a carrier that can combine several tasks into one platform to overcome hypoxia-posed tumor drug resistance arising from deep-seated lingering tumor cells.

**Figure 7:**
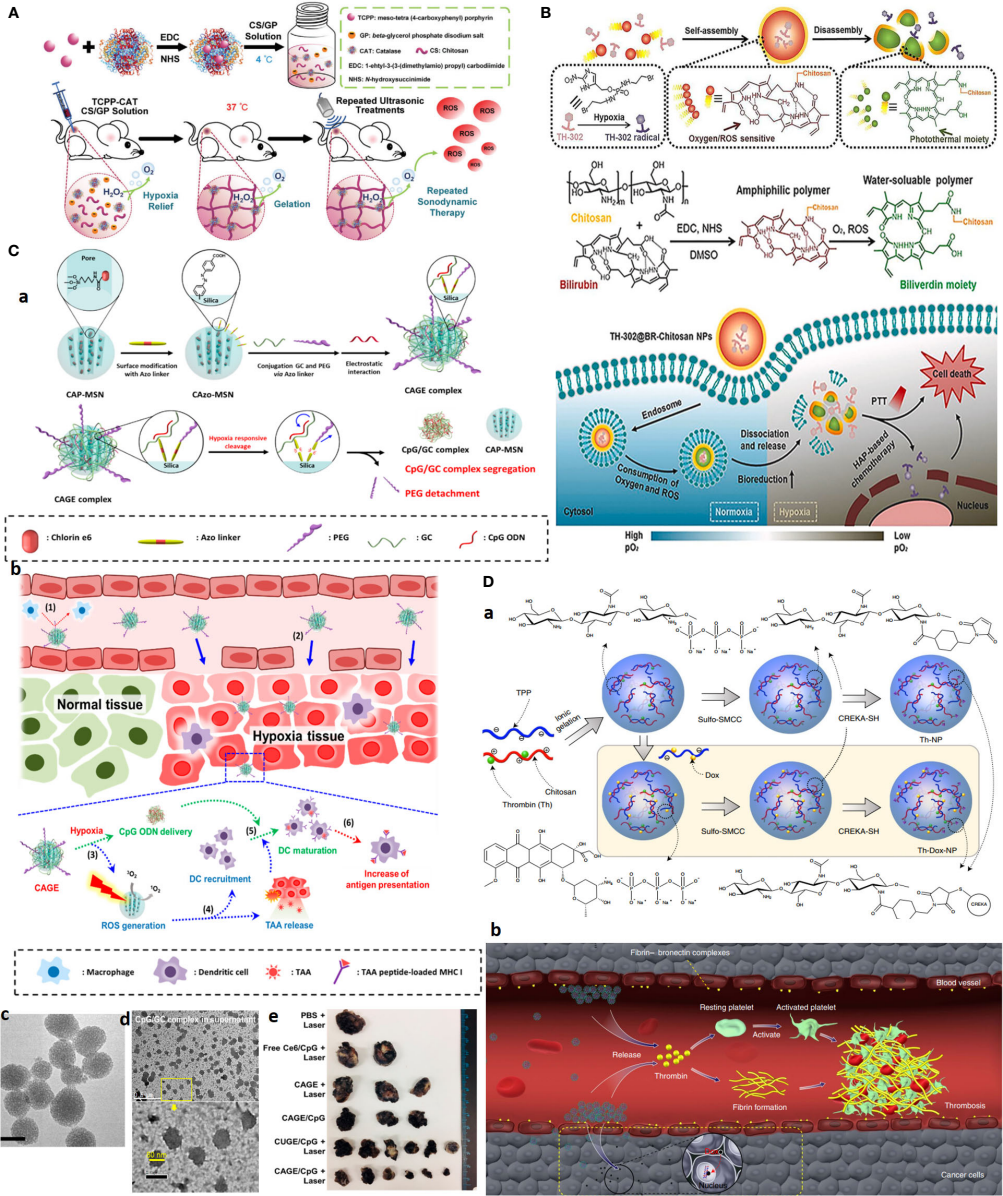
Advanced chitosan-based NPS target tumor hypoxia and result in complete tumor remission. **(A)** Hypoxia alleviation strategy. Schematics of thermo-triggered *in situ* chitosan gel formation for repeated and enhanced SDT after a single injection. Reprinted with permission from (100), Copyright (2020) Wiley‐VCH GmbH. **(B)** Hypoxia depletion therapy coupled with hypoxia-activated prodrugs (HAPs). Reprinted with permission from (109), Copyright (2021) Elsevier. **(C)** Hypoxia-responsive immunomodulation of DCs for boosted PDT coupled immunotherapy. **(a)** Schematic design of NPs, **(b)** working principle. **(c, d)** TEM images of CAGE without and with CpG ODN. **(e)** The efficacy of antitumor effects *in vivo*. Reprinted with permission from (110), Copyright (2019) American Chemical Society. **(D)** Combination of tumor infarction therapy with chemotherapy for tumor starvation therapy. **(a)** Schematic design of NPs and **(b)** their working principle. Reprinted with permission from (111), Copyright (2021) Springer Nature.

In one study, thermo-triggered *in situ* forming chitosan hydrogel is reported that is loaded with sonosensitizer meso-tetra(4-carboxyphenyl)porphine (TCPP) conjugated with catalase (CAT). The system is capable of body temperature-triggered gelation upon injection of CS-beta-glycerol phosphate disodium (GP) precursor solution into the tumor for entrapping TCPP-CAT. Then, ROS generation and deep tumor penetration can be achieved under ultrasonic treatment to effectively generate ROS as oxygen is sustainably supplied by catalase activity that decomposes endogenous H_2_O_2_ into molecular oxygen. Improving tumor oxygenation itself boosts the efficacy of repeating SDT to effectively eradicate solid tumors upon a single-dose injection (**Figure 7A**) (100).

What first appears as a problem later becomes part of the solution itself. As for hypoxia, the development of hypoxia-responsive DDSs can be envisioned using (i) hypoxia-trophic carriers such as anaerobic bacteria and stem cells, (ii) hypoxia/redox (nitroreductase enzymes)-responsive NPs using reducible cross-linking agents such azobenzene derivatives, and (iii) hypoxia-activated prodrugs (HAPs), which exclusively activate in response to oxygen degree (hypoxia/anoxia) in TME upon losing one or two electrons by activity of tumor nitroreductases to form the active drug. The significance of hypoxia responsiveness potential can be a blessing to targeted therapy of hard-to-treat tumors such as TNBC, where lack of a tumor-specific marker is a major concern.

Given that the outer proliferative tumor region is normoxic and that the optimal activity of several HAPs, such as TH302, is dependent on the extreme level of hypoxia, hypoxia aggravation by oxygen-depleting methods as described above can further improve HAPs activity. Concerning CS, an interesting oxygen-depleting self-activated nanovesicle is constructed by assembling a bilirubin (BR)-chitosan conjugate to form TH-302@BRCS NPS. BR-CS consumes O_2_, and in the presence of intratumoral-abundant H_2_O_2_, it can be oxidized into hydrophilic biliverdin (BV)-CS. In this transformation, nanovesicles disassemble to release and activate the prodrug. The inherent oxygen-consuming potential of BR-CS aggravates hypoxia for greatly enhanced HAPs-based therapy; meanwhile, BV-chitosan can be used as a photothermal agent due to its wide absorbance from visible light to the NIR region (**Figure 7B**) (109).

Similar to HAPs, TME-responsive nanocarrier systems based on hypoxia can be developed for theranostic application and allow activation of therapeutic moiety only upon localization in hypoxic tumor regions. For one, a glycol CS NPS is conjugated through a ROS-sensitive thioketal (TK) linker to a photosensitizer (PS) pheophorbide. The amphiphilic GC–TK–PNA conjugates self-assemble into self-quenching NPs. Upon reaching the ROS-rich hypoxic core, the TK bond is reduced and allows the NPS to release the PS in a photoactive form to generate potent phototoxic effects upon NIR irradiation, causing a significant reduction in the tumor volume with desirable antitumor potential (112).

Other formulations exploited a targeting moiety to cross the outer proliferative layer and then deliver hypoxia-activated nanoparticles into deep-seated tumor cells. GC cross-linked with hypoxia linker, 4-nitrobenzyl chloroformate, and decorated with folic acid (FA) can specifically release drugs under hypoxic conditions. This formulation resulted in significant growth inhibition of A549 tumor-bearing athymic nude mice upon a single injection (20 mg/kg) (113).

Hypoxia responsiveness is also harnessed for boosted cancer immunotherapy. Hypoxia-responsive Ce6 photosensitizer/adjuvant CpG codelivery system, based on glycol CS-Azo linker [4,4, PEG mesoporous silica nanoparticle (CAGE)], is developed. Under hypoxia conditions, PEG is cleaved and promotes the release of CpG/glycol CS complexes. The combined work of PDT, which induces ROS and releases tumor-associated antigens (TAAs) with CpG adjutant, enables immunomodulation by recruiting DCs and their maturation by boosting antigen presentation. Twenty-four hours after i.v. injection of NPs to the B16.F1-bearing mouse allograft model, a significant and long-lasting tumor growth inhibition plus a 100% survival rate for 28 days was observed in the CAGE/CpG group (**Figure 7C**) (110).

CREKA peptide-functionalized CS NPs are employed for combined chemotherapy with tumor-infarction therapy and chemotherapy *via* the codelivery of DOX and thrombin to tumor and tumor blood vessels overexpressing fibrin-fibronectin. Systemic administration of the CS NPs into mice bearing B16-F10 melanomas resulted in 80% complete remission for more than 45.0 days after treatment cessation (**Figure 7D**) (111).

#### 6.3.2 Immunosuppression

Crosstalk of tumor cells with various cells in the TME can inhibit host immune surveillance, resulting in tumor immune escape and tumor progression. Immune escape as a key feature of TME is shown to regulate the fate of infiltrating immune cells into the tumor. A typical example is tumor-associated macrophages (TAM), which are shifted from antitumor type M1 macrophages into pro-tumor M2 macrophages, which are related to tumor chemoresistance (3). Other mechanisms involve the secretion of immunosuppressive cytokines and host immune system dysfunction, such as excessive existence of regulatory T cells (Tregs), T-cell anergy, antigen deletion, and resistance to apoptosis. Thus, strategies to reshape immunosuppressive TME to immunostimulatory may hold promise (114) to improve the efficacy of anticancer regimes, especially cancer immunotherapy. However, direct administration of “bare” immunomodulators such as adjuvants, peptides, nucleic acids, antigens, or cytokines brings off-target toxicity and unsatisfactory efficacy due to suboptimal pharmacokinetics. Hopefully, chitosan-made NPs not only serve as natural immunoadjuvants, but also serve as a depot for antigen enrichment, allow for controlled as well as stepwise immumodualtion by controlled release of antigens, and carry immunotherapeutics such as DCs, cytokines, and other immuno adjunats to realize enhanced antitumor immune responses with reduced toxicity and side effects (115).

**Figure 8:**
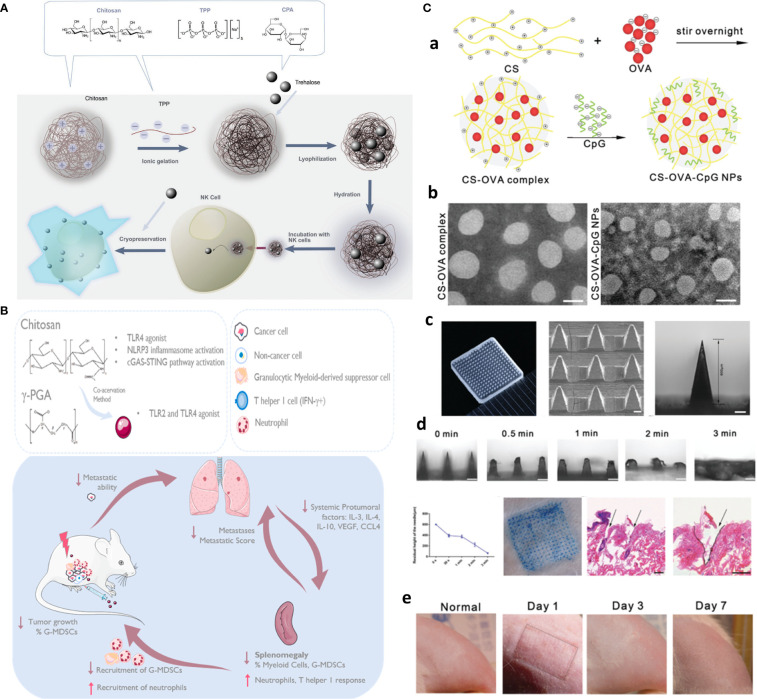
Chitosan-based platforms for active immunotherapy and metastasis inhibition. **(A)** Chitosan NPs for NK cell preservation and delivery. Reprinted from (119) under Creative Commons Attribution License 4.0, Copyright (2020) WILEY‐VCH Verlag GmbH & Co. KGaA, Weinheim. **(B)** Integration of chitosan Ch/γ-PGA NPs with radiotherapy boost immune response and elicit antimetastatic potential. Reprinted with permission from (120), Copyright (2020) Elsevier. **(C)** Chitosan NP-loaded MN patch for systemic vaccination in mice. **(a)** Schematics of synthesis of CS–OVA–CpG NPs; **(b)** TEM; **(c)** macroscopic and SEM images; **(d)** dissolution kinetics, insertion, and biocompatibility in BALB/c mouse skins; and **(e)** rat skin. Reproduced with permission from (121), Copyright (2020) Royal Society of Chemistry.

Functionalized chitosan platforms for immunotherapy involve nanogel, hydrogel, micelles, MNs (116), nanoparticles, and nanosheets with the delivery of DNA, RNA-based adjuvants as TLR agonists [e.g., CpG oligodeoxynucleotides (ODN)] (117), peptide adjuvants, as well as CAR T cell and antibody (e.g., checkpoint inhibitors) to enhance cancer immunotherapy (118). Equally, CS NPs are also used for the safe delivery and preservation of NK cells. That is, to avoid the toxic effects of cryoprotective agents, chitosan nanoparticle-mediated intracellular uptake of NK cells is replaced with dimethylsulfoxide (DMSO) to retain the potent antitumor functions of NK cells. This is the first case that delivers biocompatible nanoparticles to NK cells and shows clinical potential in manufacturing “off the shelf” safer allogeneic adoptive immunotherapies (**Figure 8A**) (119).

To circumvent challenges related to DC-based vaccination including multiple injections and elaborate *ex vivo* manipulation, CS NP is formed through ionic complexation as the next generation of vaccines. These NPs were used for delivery of OVA as a model antigen and polyinosinic–polycytidylic acid sodium salt (poly I:C) as an adjuvant to target TLR3 in endosomes. After injection, *in vivo* intracellular delivery of antigens to the DCs, and DC maturation, along with activation of antigen-specific cytotoxic CD8+ T cells, significantly greater antitumor efficacy in EG.7 and TC-1 tumor-bearing mice compared to the control was finally achieved (122). In another study, immunostimulatory chitosan/γ-PGA nanoparticle-based immunotherapy is harnessed as an adjuvant to radiotherapy in breast cancer to overcome the immunosuppressive nature, induce antitumor immunity, and control lung metastases in the 4T1 orthotopic breast tumor mouse model (**Figure 8B**) (120).

The implication of hydrogel and nanogel forms as intratumoral drug depots or post-surgery implants with immune activation potential will be discussed in the MRD section. However, an additional appealing hydrogel-based platform for immunotherapy is skin-implanted/inserted chitosan-based MN array/patches, which are capable of simultaneous sampling, injection, and sustained release of adjunct/immunostimulant agents, and given the flexibility and stretchability as wearable devices, there is no concern for their displacing or deformation upon body movement (7, 123).

For one, a needle-free injection MN array is developed with PVP on the top layer and fast-dissolving (3 min) chitosan NPs coloaded with OVA and CpG as an adjuvant to evoke systemic immune responses in mice (**Figure 8C**) (121). Likewise, sodium hyaluronate (SH) as tip and chitosan as needle base afforded long-term immunization using a single-shot intradermal immunization. Biodegradable chitosan base remained in the derm and afforded long-term OVA release for 4 weeks, while fast-dissolving SH acted as the promising dose by rapid releasing of encapsulated OVA, resulting in enhanced cytotoxic immunity as well as higher and more durable antibody response (124).

An additional immune-based strategy aims to reshape/reprogram immunosuppressive TME and tumor-promoting stroma into an immune-activated state, for example, by reprogramming protumor TAM-2 into antitumor TAM-1 (3), or targeting CAFs as attempted by dual targeting of CAFs and tumor cells through angiotensin II type I receptor (AT_1_R) overexpressed on both CAFs and tumor cells using pathological inspired micelles (74). Additional biomaterial-based strategies that aim to reshape immunosuppressive TME in a spatiotemporal manner for enhanced cancer immunotherapy are reviewed in Ref (125).

#### 6.3.3 Cancer stem cells

CSCs are rare subpopulations of cancer cells that are highly tumorigenic and causative of cancer relapse and metastasis owing to their high capability of initiating and/or reinitiating tumor regrowth. Hostile TME conditions such as hypoxia and external assaults such as chemotherapy treatment can promote stemness and stem cell plasticity. CSCs are hard to treat and develop drug resistance owing to their specific characteristics, including clonal evolution and heterogeneity, dormancy, enhanced DNA repair, overexpressed drug efflux pumps, and overexpression of antiapoptotic proteins (1, 126).

Up to now, CSC targeting and studying using 3D modeling systems remain an untraceable challenge as tumor cells tend to switch from EMT to MET, losing and gaining stemness behaviors. In addition, high-throughput screening of 16,000 compounds identified only ∼0.2% compounds with selective toxicity for breast CSCs (127). This call for advanced strategies is effective not only against tumor bulk but also for the elimination of residual cells, including CSCs and CSLCs.

**Figure 9:**
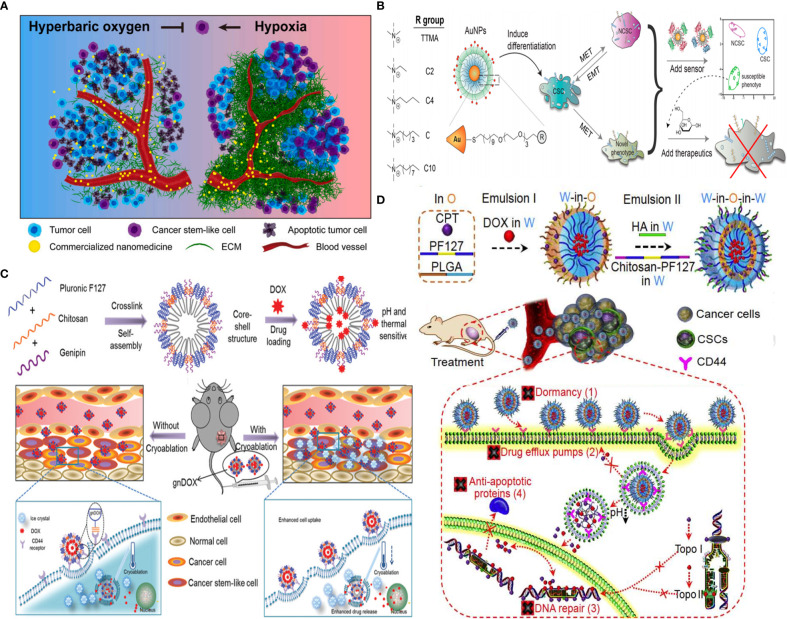
Strategies for CSC targeting and elimination. **(A)** Hyperbaric oxygen therapy. Reprinted with permission from (92), Copyright (2021) Elsevier. **(B)** NP surface modification from hydrophilic (TTMA) to highly hydrophobic (C10) tested, with C6 AuNP robustly promoted CSC differentiation by reducing stemness marker. Reprinted with permission from (128), Copyright (2020) American Chemical Society. **(C)** Multimodal system combines chemo- and cryoablation therapy. Reprinted with permission from (129), Copyright (2021) Elsevier. **(D)** Receptor-mediated dual drug combination strategy. Reprinted with permission from (130), Copyright (2021) Royal Society of Chemistry.

Generally, CSC targeting approaches are based on several strategies: (i) hypoxia reduction as attempted by hyperbaric oxygen therapy to improve oxygenation and enable deep penetration of commercialized nanomedicine for CSC eradication (**Figure 9A**) (92), (ii) CSC targeting by CSC-specific markers decorated with NPs (e.g., CD44 marker) (17), (iii) CSC “differentiation” strategy, using reprogramming factors/agents) (131) or “defanging” CSCs through nanoparticle surface engineering (**Figure 9B**) (128), and (iv) “cocktail” therapy (132) with the combination of multiple drugs (**Figure 9D**) (130), or multimodal systems combining different strategies (e.g., chemotherapy and PDT for enhanced ROS generation) (129) (**Figure 9C**).

Chitosan has been used for CSC targeting, and chitosan-based scaffolds (membrane) were also used as promising biomimetics for the CSC niche to study their behavior. Cultured colon and HCC cells on chitosan membranes can develop stemness properties with increased cell motility, quiescent population, drug resistance, self-renewal capacity, and the expression levels of stemness and CSC marker genes, such as OCT4, NANOG, CD133, CD44, and EpCAM (133).

Also, single-cell mass cytometry revealed that DOX therapy of MDA-MB-231 cells increases stemness properties of their spheroids partially *via* αvβ3 integrin overexpression. An RGD-modified CS NP promoted chemotherapy efficacy against these CSCs (134). Rather than using CSC markers, CS, owing to pH responsiveness and inherent property to target CD44^+^ CSCs, but not CD44^+^ normal stem cells, was decorated on the surface of pluronic F127 consisting of hydrophilic PEG and the more hydrophobic polypropylene glycol (PPG). After systemic injection of developed 20-nm nanoparticles, they accumulated in the tumor through the EPR effect and afforded endolysosomal pH-dependent DOX delivery six times more efficient than DOX delivery alone to eliminate tumor-reinitiating CSLCs in 3D mammospheres and reduced the size of tumors in an orthotopic xenograft tumor model with no evident systemic toxicity (17). Likely, another formulation consisting of HA-decorated thermal and pH-responsive nanoparticles of pluronic F127, PLGA, and CS is attempted for targeted codelivery of DOX and irinotecan to eliminate CSLCs where HA is used for active targeting of CD44+ CSCs to reduce their drug resistance due to dormancy, F127 for thermal responsiveness, and chitosan for acidic pH-triggered drug release; DOX and irinotecan-loaded NPs were used to inhibit the activity of topoisomerases II (DOX) and I (irinotecan), respectively, to fight CSC drug resistance associated with their enhanced DNA repair and antiapoptosis, while minimizing drug efflux from CSCs due to drug nanoformulation. This resulted in targeting and efficient breast CSC eradication both *in vitro* and *in vivo* with up to ~500 times of enhancement compared to two drugs without nanoformulation (**Figure 9C**) (130). Moreover, core–shell nanoparticle formation is achieved by self-assembly of genipin cross-linked pluronic F127-chitosan nanoparticles (GNPs) and the efficient encapsulation of DOX combined with cryoablation therapy. DOX release can be triggered by thermal and pH stimuli upon deposition of a cryogenic agent at the target tumor site, allowing for synergistic effects and chitosan-mediated CSC-like targeting of chemoresistant mammary tumors (**Figure 9D**) (129).

#### 6.3.4 Circulating materials: CTCs and exosomes

TME-derived circulating materials, including cell-free DNA (cf-DNA), circulating tumor cells (CTCs), and exosomes, are key contributors to MRD and occult metastasis. Though they can serve as invaluable biomarkers for early cancer detection and metastasis status, capturing (detection) and analysis of these rare and heterogeneous elements from trace biological samples in clinics are a challenge (3). Hopefully, properties of chitosan such as inherent affinity for epithelial cell adhesion and ease of functionalization with CTC-specific markers such as anti-epithelial cell adhesion molecule (anti-EpCAM) antibody inspired the devising of chitosan-based trapping systems with the capability to be integrated with microfluidic chip to bring extraordinary potentials with simplicity, high purity, sensitivity, and viability (nondestructive release), and in a label-free fashion (135).

**Figure 10:**
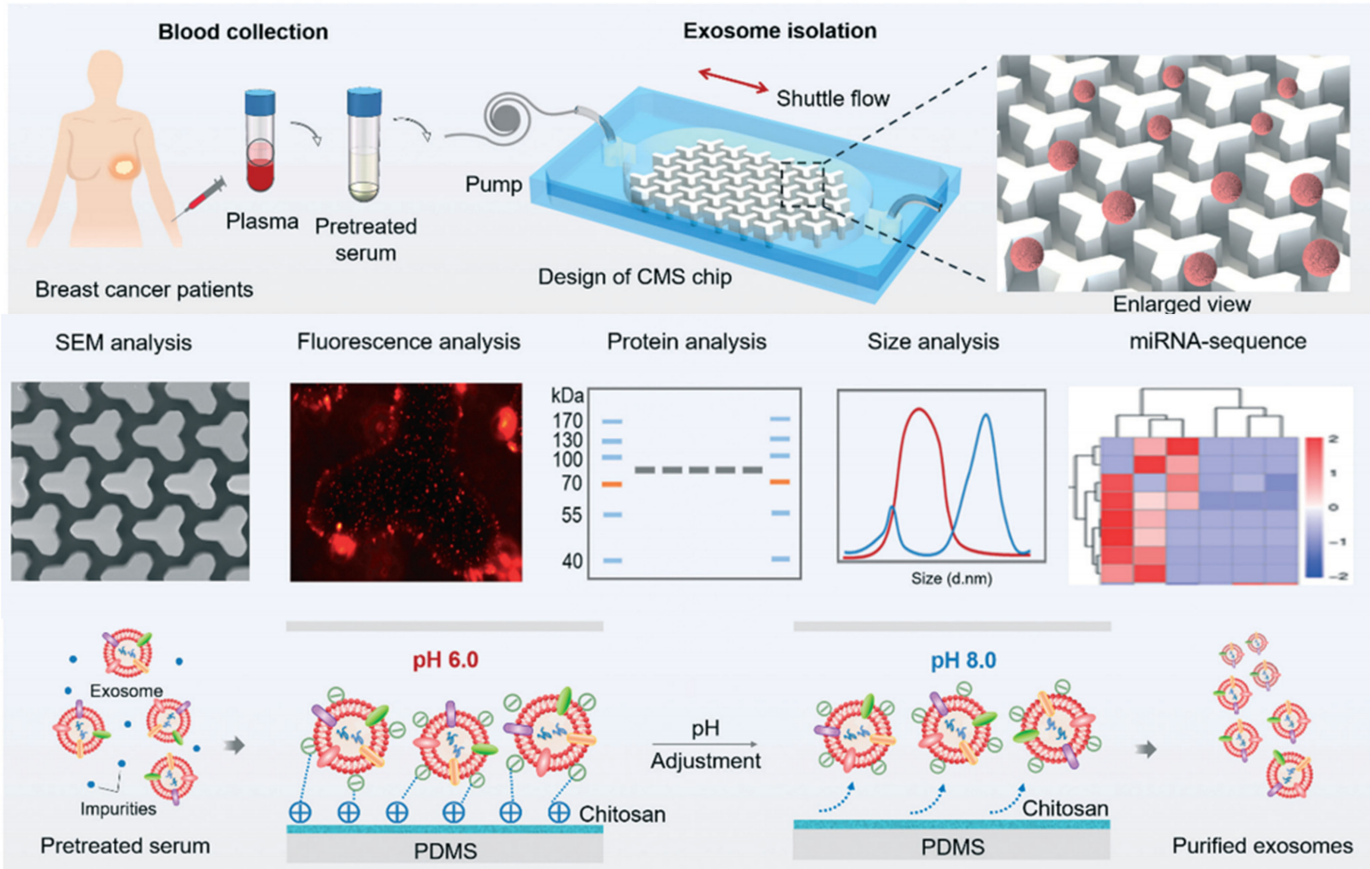
CS-based liquid biopsy for interrogation and elimination of circulating materials. Chitosan-based microfluidic platform for tumor-derived exosome isolation and analysis. Reprinted from (135), Copyright (2021) Royal Society of Chemistry.

In one preparation, a simple and fast method for isolation of circulating exosomes is reported, which integrates chitosan electrostatic adsorption, micro-patterned substrates, and microfluidic shuttle flow control to enable the capture/release of circulating exosomes from trace clinical blood samples (10 μl) with more than 90% purity and above 84% high RNA recovery ratio within 15 min, outperforming traditional ultracentrifugation methods (**Figure 10**) (135). Likewise, a fibrous mat using HA-functionalized electrospun CS nanofiber (CNF) is generated and embedded in a microfluidic chip to enable the specific capture and release of CD44-overexpressing CTCs. Modification of electrospun CNFs with zwitterion carboxyl betaine acrylamide (CBAA) elicits hemocompatibility and an excellent antifouling property, and can capture A549 human lung cancer cells with an efficiency of 91% at a flow rate of 1.0 ml/h and their non-invasive efficient release (90%) upon the addition of glutathione for 40 min. In tested clinical blood samples, the presence of MRD in 5 of 5 breast cancer patients and 9 of 10 non-small-cell lung cancer patients was detected with high sensitivity down to single cell to 18 CTCs per milliliter of blood (136). Similar work is also attempted using anti-EpCAM antibody functionalized hydrogel-chitosan nanofiber with a capture yield of 79.9% in artificial blood samples (137).

## 7 Tumor-penetrating chitosan NPs for the treatment of minimal residual disease

Chitosan NPs afford crossing numerous biological barriers; an important application is the treatment of relapsed tumors and metastasis. The latter category involves injectable irregular wound-filing hydrogels employed for treatment of post-surgery recurrence and wound tissue reconstitution post-surgery. To achieve this, hydrogels are injected either into or in the vicinity of the tumor without the invasive procedure of tumor surgery, which triggers bleeding and metastasis.

### 7.1 Intratumoral injectable hydrogel

**Figure 11:**
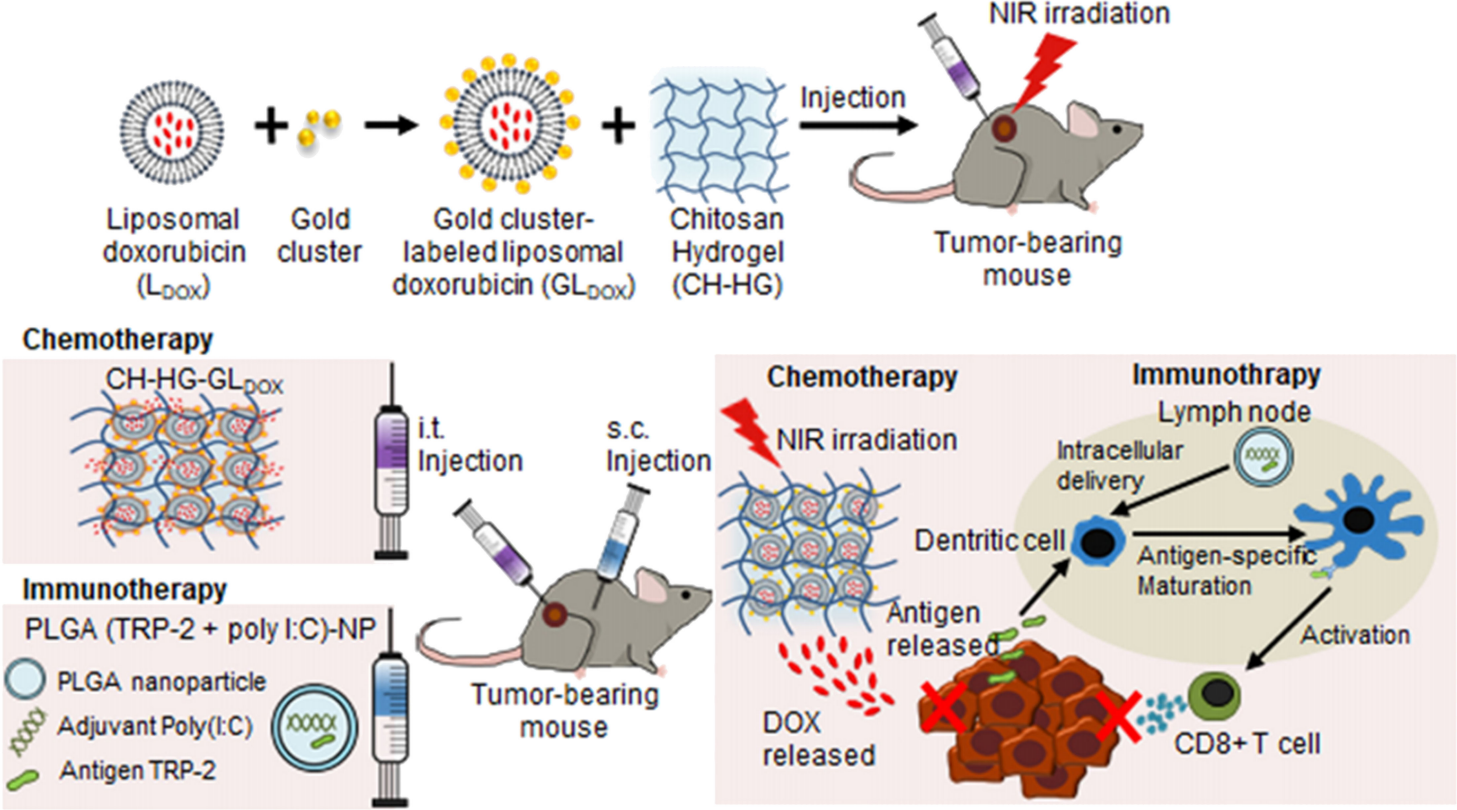
Local injectable chitosan serves as a drug depot to control tumor relapse. Intratumoral injectable chitosan nanoformulation for combined chemo- and immunotherapy of cancer. Reprinted with permission from (139), Copyright (2021) Elsevier.

Serving as a long-lasting drug depot, direct hydrogel injection into the tumor site as preformed nanoparticles or *in situ* forming hydrogels is ideal for local treatment of chronic diseases such as inflammation and cancer, with preferential, controlled, and on-demand capacity for drug release. To this, a highly efficient chimeric antigen receptor T lymphocyte (CAR T) delivery is developed for local immunotherapy of recurrent cancer. In this line, injectable thermosensitive CS-PEG hydrogel is used for loading ganglioside GD2-specific CAR T cells and their long preservation to express a recombinant immune-enhancing cytokine, interleukin-15, as a self-growth factor support for promoting T-cell proliferation, activation, and survival. CAR T cells can preserve vision as they improve antitumor effects to control retinoblastoma, with 60% complete remission and being tumor-free up to day 70 in mice (138). Also, CS hydrogel serves as a drug depot for the delivery of gold clustered liposomal DOX. CH-HG-GLDOX can be directly injected into tumor tissue without a surgical procedure and release DOX upon NIR irradiation. Furthermore, CH-HG-GL_DOX_ and poly(D,L-lactide-co-glycolic acid) nanoparticle-based vaccines increased cytotoxic CD8+ T-cell immunity, leading to enhanced synergistic chemotherapy and immunotherapy efficacy (**Figure 11**) (139).

In another case, an injectable and photo-responsive drug delivery antibacterial hydrogel (CP@Au@DC_AC50) is reported for uveal melanoma (UM) treatment. Mechanical strength and PTT are afforded due to the incorporation of gold nanorods and resulted in thermosensitive gel-sol transformation to release the gene-targeted drug DC_AC50 on demand in response to low-density NIR light. This multifunctional therapeutic platform affords NIR light-triggered gene-targeted therapy/PTT/antibacterial potential in the orthotopic model of UM as intraocular tumors (**Figure 2A**) (65). Electrospun nanofibers composed of QCS, polyvinyl alcohol (PVA), and curcumin are fabricated with excellent anticancer activity against recurrent 4T1 breast cancer cells and antimicrobial performance against *Staphylococcus aureus* and *Escherichia coli* bacteria (140).

### 7.2 Post-surgery injectable/implanting hydrogel

After tumor resection, chitosan-based precursors can be injected into the wound to form a hydrogel. Implants or surgical glues can also be used; however, unlike *in situ* forming gels, they cannot fill irregular wounds (141). Sol-gel transition can be triggered by internal (e.g., temperature) or external (e.g., light) triggers that allow *in situ* gelation and thus entrapping of various cargoes and their long-term retention for a sustained release. At the same time, localized yet augmented therapeutic effects with off-target systemic effects can be envisioned (9). Furthermore, controlled specific cargo release from gel in response to TME changes, such as pH, hypoxia, specific gene/protein expression changes, or externally induced by light (ultrasound waves using SDT, PTT, and PDT), can be realized, which all rely on ROS generation to eliminate residual tumor cells (63, 141–143).

Nanocomposite made of CS has wound healing, antimicrobial, adhesive, and tissue regenerating potentials. The multi-functionality of advanced CS hydrogels/fibers combined with other approaches such as chemotherapy and particularly immunotherapy (e.g., anti-PDL antibody) allows for synergistic therapy to effectively detect and kill residual/regenerating tumor cells (141) (142).

Bioinspired hydrogels are another advanced and highly promising tool for clinical translation (123). In this line, inspired by catechol groups found in marine Mussel foot protein, bioinspired adhesive chitosan can be fabricated to enable additional functionality, which is capability for tight wet adhesion if it is meant to be used in surgical trauma. Another consideration is anti-inflammatory and antimicrobial potentials, as adhesive hydrogels are prone to bacterial infection while surgery wound trauma itself triggers the inflammatory condition and, thus, tumor recurrence (63, 142).

**Figure 12:**
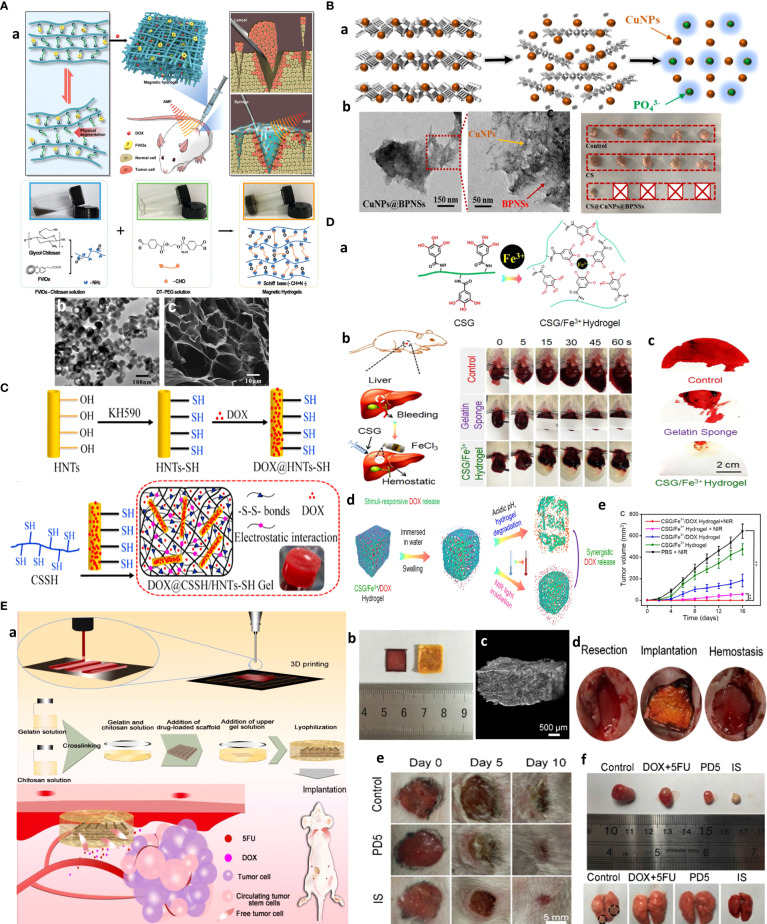
Post-surgery chitosan injectable gels/implanted scaffolds for treating recurrent cancer and MRD. **(A)** Magnetic hydrogel for the treatment of breast cancer metastasis. **(a)** Schematics of design (top) and transformation mechanism (bottom) of GC-ferrofluids to form a magnetic hydrogel. **(b)** TEM and **(c)** SEM images of porous magnetic hydrogels. Reprinted from (141) with permission, Copyright (2019) WILEY‐VCH Verlag GmbH & Co. KGaA, Weinheim. **(B)** Injectable biodegradable hydrogel for multiple tumor and recurrent cancer therapy. **(a)** Schematic for the degradable process of the CS@CuNPs@BPNSs hydrogel. **(b)** TEM image **(c)** and efficacy of CuNPs@BPNSs for complete tumor remission in the lung cancer recurrence model. Reprinted from (142) with permission, Copyright (2021) Elsevier. **(C)** Thermosensitive injectable halloysite-chitosan hydrogels as drug carriers for inhibition of breast cancer recurrence and tissue reconstruction. **(a)** Synthesis procedure for thiolated halloysite nanotubes (HNT) for DOX loading and hydrogel formation by cross-linking with chitosan through SS bonds and electrostatic interactions. Reprinted from (20) with permission, Copyright (2021) Elsevier. **(D)** Anti-inflammatory and antibacterial catecholic chitosan hydrogel for rapid surgical trauma healing and subsequent prevention of breast tumor recurrence. **(a)** Synthesis route of CSG/Fe^+3^/DOX hydrogel by Fe^+3^ chelation of chitosan (CS)–gallic acid (GA) and DOX loading. **(b)** hemostatic potential in a model of rat liver bleeding, **(c)** typical bloody filter papers. **(d)** pH/NIR-responsive DOX **(e)** Effective 4T1 tumor recurrence of the CSG/Fe3+/DOX hydrogel. Reprinted from (63) with permission. Copyright (2020) Elsevier. **(E)** 3D printed intelligent scaffold (IS) stops bleeding and adsorbs CTC after surgery to prevent recurrence and distal metastasis of breast cancer. **(a)** Electro-hydrodynamic jet 3D printing process of drug-loaded chitosan–gelatin (CG) scaffold, **(b)** macroscopic, **(c)** microscopic, **(d)** SEM images of implanted IS after 30 days, **(e)** potential of IS for wound healing and closure compared to gauze (control) and PLGA-DOX-5FU (PD5) scaffold, **(f)** potential of IS for recurrent cancer and inhibiting breast cancer lung metastatic activity. From (144), Copyright (2020) Under Creative Commons Attribution License 4.0. ivyspring.

As an example of injectable CS-based hydrogels, the fabrication of a novel magnetic hydrogel is reported, which incorporates very low amounts (0.6 mg ml^−1^ compared to SPIO-magnetic hydrogels) of ferromagnetic vortex-domain iron oxide (FVIOs) grafted on the surface of GC and then mixed with DT-PEG solution, which forms coupling–uncoupling imine bonds between CS chains, affording dynamic hydrogel. FVIO-functionalized hydrogel (F-MH) possesses functional adaptability to TME as it imparts sufficient heating capacity, rapid sol (25°C)-gel (37°C) transformation (~400 s gelation time), self-conformal ability to seep into and conform to small gaps of the wounded area, self-healing, biodegradation, heating potential, and pH-responsive controlled release of DOX to effectively prevent breast cancer recurrence *in vivo* (**Figure 12A**) (141).

In another study, CS hydrogel composed of black phosphate nanosheets (BPNSs) and *in situ* grown copper nanoparticles (CuNPs) was developed. As an injectable gel, CS@BPNSs@CuNPs form a quick spongy-like state when the temperature is raised to 37°C and possess a 24.98% blood clotting index, a 91% antimicrobial effect against *E. coli* and *S. aureus* through ROS generation, and 10% tumor recurrence. Combined with aPD-L1-based immunotherapy, or PTT, this biodegradable injectable *in situ* forming hydrogel is promising for postoperative trauma and combating multiple primary tumors, including subcutaneous lung tumor and second primary HCC model and ectopic *in situ* glioblastoma model (**Figure 12B**) (142). Likewise, an injectable biocompatible thiolated chitosan (CSSH) hydrogel is fabricated, composed of DOX-loaded thiolated halloysite nanotubes (HNTs) with good mechanical properties. Further CSSH cross-linking permits gel formation of DOX@CSSH/HNTs-SH. This hydrogel allows pH responsiveness DOX release to inhibit the recurrence and repair the defected tissue after tumor resection (**Figure 12C**) (20). Mussel-inspired catecholic-Fe^3+^ chitosan-gallic acid (CSG) hydrogel is formulated to well-fill the tumor-resected cavity with considerable anti-inflammatory and wet-adhesion ability, surgical trauma healing, photothermal agent (catechol-Fe^3+^ moiety), and subsequent prevention of local tumor recurrence benefiting from the combined work of photothermal therapy (rising tumor Tm to ~46°C) and chemotherapy (pH and NIR-induced DOX release) in the 4T1 breast cancer model in mice (**Figure 12D**) (63).

Hydrogel implants are used to control MRD. Local delivery of lentinan, a stemness-depleting agent, is afforded by fabrication of a sponge-like LNT/chitosan composite to be implanted and used for sustained local drug delivery and long-term tumor relapse inhibition of postoperative breast cancer *in vitro* and *in vivo* (145). Likewise, cisplatin-loaded hyaluronate-chitosan placed as intrapleural polymeric films containing cisplatin inhibited the local recurrence of malignant pleural mesothelioma in a rat tumor model (146). Finally, 3D printed intelligent poly(lactic-co-glycolic acid), gelatin, and chitosan implant loaded with anticancer drugs 5-FU and DOX is prepared. This intelligent scaffold (IS) renders hemostatic function and accelerates the wound closure. IS allows on-demand drug release in response to pH, can adsorb residual cells (e.g., CTCs), and prevents recurrence and distal metastasis of skin-implanted MDA-MB-231 breast cancer with 80% survival rate in mice. The *in vivo* antitumor ability of the IS was observed for up to 30 days with good antitumor activity in the absence of lung metastases compared to controls (**Figure 12E**) (144).

## 8 Chitosan in the clinic: Concluding remarks and future perspective

Natural products and nature-inspired biomaterials are appealing for biomedical uses. Among the clinic’s biomaterials and delivery systems are vesicular particles, mostly liposomal and polymeric (pegylated) forms as FDA-approved nanodrugs. We also recently proposed the bioinspired hydrogel-based products as next-generation biomaterials with a high chance for translation into the clinic (123). Concerning chitosan, as a natural (semi-natural) product, the many inherent properties besides its simplicity, functionality, and full addressability to be combined with other materials to make multifunctional systems can extend its potential for numerous applications for drug delivery, having different forms such as nanogels, nanofibers, hydrogels, vesicular particles for codelivery of hydrophobic and hydrophilic drugs, and multimodal and important biochips to be used for interrogation of circulating materials in plasma samples of cancer patients, enabling personalized medicine.

With tumor heterogeneity as the main hurdle for efficient nanodrug delivery and TME-derived numerous biological barriers, chitosan-based drug formulations have shown promising results. Most lycol forms of chitosan are best for targeting tumor heterogeneity, particularly lymph node metastasis targeting, with host immune cell evoking antimetastasis potential. Further combining different chitosan derivatives, each possessing a specific function into a single formulation, can uncouple unprecedented features to tackle tumor heterogeneity and cross several barriers at once. For example, mucoadhesion using fluorinated chitosan coating may lead to development of oral vaccines for transepithelial transport and cloak NPs for longer circulation. At the same time, oligosaccharide forms can provide stem-cell-like population targeting. Advances in chitosan platforms as implantable trapping devices for capturing and releasing CTCs through their adhesive potentials to epithelial cells are another appealing potential as a label-free diagnostic and prognostic method.

The main applications of chitosan are cosmetic, dietary supplement, teeth implant, drug excipient, wound healing dressing, and drug delivery. The FDA approves chitosan for wound dressings and drug and gene delivery in humans (13). There are a total of 117 records on clinicaltrials.gov using “chitosan”, 13 records for “chitosan” and “wound healing”, and only six clinical trials searched using “chitosan” and “cancer” as keywords (**Table 1**). The first two clinical trials were for chitosan used as an anticancer agent by reducing levels of AGE and as a pain killer. Concerning chitosan-based cell culture models, a recent project is recruiting lung cancer patients to evaluate the correlation of the cell detachment ratio on pH-responsive chitosan with the overall survival as a diagnostic and prognostic indicator by flow cytometry analysis of tumor (stem-like) cell-specific markers expressed by isolated cells. Acting as an adhesive barrier, Mediclore^®^, a mixed solid of poloxamer, gelatin, and chitosan, is undergoing tests for its safety and antiadhesive effect for post-axillary dissection surgery in breast cancer patients. The mean difference in range of motion (ROM) and pain will be compared among patients undergoing standard treatment for surgery and those in whom Mediclore^®^ was injected into the surgery site and closed with a suture. In another project taking 58 weeks, local photothermal laser-based immunotherapy as a trigger for total antigen release from whole cells was combined with 1% immunostimulant glycated chitosan (GC) injection (1 ml injection in and around laser-irradiated tumor site) as an adjutant, for the activation of APCs to take up tumor antigens and activate and forward patient’s tumor-specific systemic immune response, for residual/metastatic remaining tumor(s). This also involved low-dose cyclophosphamide, and the outcome was based on overall response rate and overall survival. Likewise, a similar ongoing project initiated in 2019 also aims to treat advanced solid tumors using photothermal ablation immediately followed by intratumoral injection of IP-001 (1% *N*-dihydro-galacto-chitosan, Immunophotonics Inc.) to turn “cold” into “hot” tumors by heat-mediated tumor antigen-releasing and IP-001-triggering systemic tumor-specific immune response to eliminate residual primary and metastatic tumor cells far from the treated area (also known as abscopal effect).

**Table 1:** Chitosan clinical trials for advanced recurrent cancer therapy.

Title	Condition	Country	Phase	NCT number	Date
Study of chitosan for pharmacologic manipulation of AGE (advanced glycation end products) levels in prostate cancer patients	Prostate cancer	United States	Phase 1–2	NCT03712371	2019–2021
Comparison of oral morphine versus nasal ketamine spray with chitosan in cancer pain outpatients	Cancer pain	Switzerland	Phase 3	NCT02591017	2015–2018
Evaluation of the cell detachment ratio on pH-responsive chitosan as a prognostic factor in lung cancer	Lung cancer	Taiwan	Unknown	NCT04218188	2020–ongoing
Antiadhesive effect and safety of a mixed solid of poloxamer, gelatin, and chitosan (Mediclore^®^) after axillary dissection for breast cancer	Breast cancer	Korea	Phase 3	NCT02967146	2017–ongoing
A randomized clinical trial evaluating the use of the laser-assisted immunotherapy (LIT/inCVAX) in advanced breast cancer	Breast cancer	Peru	Phase 3	NCT03202446	2017–2018
Intratumoral injection of IP-001 following thermal ablation in patients with advanced solid tumors	Advanced solid tumors	Switzerland	Phase 1–2	NCT03993678	2019–ongoing

Together, these experiments show the potentials of chitosan for solid tumor therapy in the context of relapse and resistance. Only chitosan or its derivatives were potent; thus, there is still room to make important discoveries using various nanoformulations in the preclinical setting to be tested in the clinic. Among those, we envision that bioinspired multifunctional formulations, especially hydrogel-based formulations, are amenable as drug depots to afford long-term and pH-responsive drug release for the elimination of residual tumor cells, wound healing after tumor resection, and multimodal theranostics in an imaging-guided “hunting and killing approach” of primary and remaining tumors. Additionally, chitosan formulations can also be prepared for systemic injections and treatment of hematological cancers. Further improvement in chitosan derivatives, synthesis approaches, molecular characteristics, rational modification/functionalization, control over molecular weight and the degree of modification, and incorporation of multi-stimuli-responsive moieties enables the translation of the full potential of chitosan to clinical products.

## Author contributions

HM, MJ and RJ-E have written the original draft, MAAA and MSh helped with data collection, ZA and RJ-E supervised and edited the final draft.

## Acknowledgments

RJ-E is supported by NIMAD (grant number: 978679) and Tabriz University of Medical Sciences, Tabriz, Iran (grant number: 65568).

## Conflict of interest

The authors declare that the research was conducted in the absence of any commercial or financial relationships that could be construed as a potential conflict of interest.

## Publisher’s note

All claims expressed in this article are solely those of the authors and do not necessarily represent those of their affiliated organizations, or those of the publisher, the editors and the reviewers. Any product that may be evaluated in this article, or claim that may be made by its manufacturer, is not guaranteed or endorsed by the publisher.
